# Genome‐Wide Association Study in European Beech ( *Fagus sylvatica* L.) for Drought Stress Related Traits

**DOI:** 10.1111/ppl.71120

**Published:** 2026-09-22

**Authors:** Mila Tost, Ourania Grigoriadou‐Zormpa, Selina Wilhelmi, Timothy Beissinger, Mehdi Ben Targem, Markus Müller, Henning Wildhagen, Alexandru Lucian Curtu, Oliver Gailing

**Affiliations:** ^1^ Division of Plant Breeding Methodology University of Göttingen Göttingen Germany; ^2^ Forest Genetics and Forest Tree Breeding, University of Göttingen Göttingen Germany; ^3^ Center for Integrated Breeding Research (CiBreed), University of Göttingen Göttingen Germany; ^4^ Heritable Agriculture Mountain View California USA; ^5^ Faculty of Resource Management HAWK Göttingen Germany; ^6^ Department of Silviculture Transilvania University of Brasov Brasov Romania

**Keywords:** *Fagus sylvatica*, GWAS, natural population, polygenic selection, population genetics, quantitative genetics

## Abstract

Genome‐wide association studies (GWAS) aim to identify loci associated with different traits, which could be evaluated in subsequent studies and later be prioritized in individual and seed stand selection. In trees, traits are measured at maturity or are only available after a long time, and juvenile‐mature correlations are often unknown. Therefore, it is a promising approach to study natural populations directly. Data were collected from approximately 100 adult beech trees per stand in five locations in the Postăvarul Massif, South‐Eastern Romanian Carpathians (Carpații de Curbură) along an altitudinal gradient associated with precipitation and temperature. We performed GWAS on 610,678 single‐nucleotide polymorphisms (SNPs) using PLINK and Genome‐wide Efficient Mixed Model Association (GEMMA) to identify SNP markers associated with drought‐stress‐related traits. Even though this study used a natural population, we found very interesting associations while accounting for environmental heterogeneity along the altitudinal gradient. A total of 88 markers were associated with stomatal density. Seventy‐two markers are located on chromosome 10 in a region spreading from ~5.48 to 13.67 Mb. We found five gene annotations in this region that are thought to play a role in controlling stomatal density in *Arabidopsis*. All markers within this region exhibit similar allele frequencies, which indicate a connection to stomatal density and the altitudinal gradient. This entire region may be under joint selection for stomatal features. Our research provides insights into the genetic architecture of various drought‐stress traits in the studied population.

## 1 Introduction

In forest tree improvement, different tree stands exposed to specific environmental conditions are sampled and tested in provenance trials (Langlet 1971; Rellstab et al. 2015). Adult trees are later tested because growth and quality trait measurements from juvenile trees are not reliable (Neale and Kremer 2011). In the face of climate change, provenance trials are also used to make decisions on the transfer of forest reproductive material to identify appropriate material at the population level with desirable wood quality and growth traits (Rellstab et al. 2015; Cortés et al. 2020). Proper evaluation of the performance of stands can take between 20 and 25 years in long‐living woody perennial species like European beech ( *Fagus sylvatica* L.) (Neale and Kremer 2011), and only then does the seed and seedling production start (Neale and Kremer 2011; Cortés et al. 2020). Therefore, it is of great interest in forest tree improvement to determine the genetic basis of traits to predict the potential for success of selection before selection starts (Cortés et al. 2020). Furthermore, identifying candidate gene markers associated with specific traits is desired, as they could be utilized in forest improvement programs (Neale and Kremer 2011; Rellstab et al. 2015; Cortés et al. 2020; Müller et al. 2024).

Genome‐wide association studies (GWAS) aim to identify associations between markers and phenotypic traits of interest, for example, wood yield, wood properties, resistance to diseases, or abiotic stresses (Visscher et al. 2017; Neale and Kremer 2011). Alternatively, other approaches for the detection of markers or quantitative trait loci (QTL) exist, which comprise QTL mapping (Hall et al. 2016). In the absence of reference genome data, this approach was widely used in forestry research. However, with the increasing availability of reference genomes for tree species, genome‐wide association studies (GWAS), also known as association mapping, have become more common (Hall et al. 2016; Ingvarsson and Street 2011). QTL mapping requires a segregating mapping population, which is often available in poplar species (Jorge et al. 2005) but often not available in non‐model tree species such as beech.

GWAS return peaks of the “genomic landscape” of adaptation (Rellstab et al. 2015), but according to the “infinitesimal model” of Fisher (1918), quantitative traits are controlled by many small‐effect loci. Several methods have been introduced based on Fisher's infinitesimal model (Berg and Coop 2014; Zeng et al. 2018), such as $\hat{G}$ (Beissinger et al. 2018) to test for polygenic selection. $\hat{G}$ follows the model of Wright (1931), in which, under selective pressure, alleles with positive effect sizes increase in frequency over generations. In the $\hat{G}$ test, all loci are considered together to retrieve a signal of polygenic selection (Beissinger et al. 2018). $\hat{G}$ is calculated by using an estimation approach from genomic prediction (Beissinger et al. 2018; Mahmoud et al. 2023). But instead of predicting phenotypes or breeding values, $\hat{G}$ uses the estimated marker effects and combines them with allele frequency differences calculated between different groups that have encountered a selective pressure (Beissinger et al. 2018; Mahmoud et al. 2023). $\hat{G}$ offers an advantage over genomic prediction by providing information on the adaptive or selective pressures that have shaped a trait and can even indicate the direction of selection. This allows us to determine whether a trait has been subject to negative or positive selection (Beissinger et al. 2018; Mahmoud et al. 2023). Additionally, $\hat{G}$ can be used to identify signals of polygenic selection when effect sizes are too small to pass the significance threshold of a GWAS (Beissinger et al. 2018; Mahmoud et al. 2023). Therefore, $\hat{G}$ is a promising new approach to test for polygenic selection under environmental selective pressures (Beissinger et al. 2018). While studies in crop species have shown that $\hat{G}$ successfully identify signals of polygenic selection (Mahmoud et al. 2023; Morales et al. 2021), no applications of $\hat{G}$ to forest tree species have been reported.

European beech is one of the most important forest tree species in Europe, accounting for the highest percentage of broadleaf growing stock on the continent (Houston Durrant et al. 2016). It is a shade‐tolerant broad‐leaved tree in Europe and is used for furniture, musical instruments, pulp for paper production and firewood (Houston Durrant et al. 2016). European beech was considered tolerant to episodic droughts (Hartmann et al. 2022). However, repeated drought events from 2018 to 2020 had a significant impact on European beech. Recent studies report that the species is adversely affected by drought stress driven by climate change (Hartmann et al. 2022; Martinez del Castillo et al. 2022). Various validated climate change prediction models indicate declines in growth across large regions of Europe (Martinez del Castillo et al. 2022). Beech trees are dying in large numbers in drought‐affected regions. The summer climate in southern Europe, including the Balkans, may resemble the future climate of Central Europe (Martinez del Castillo et al. 2022; Baumbach et al. 2019). Thus, beech stands from southern Europe could serve as genetic resources for Central Europe (Martinez del Castillo et al. 2022; Baumbach et al. 2019). To our knowledge a limited number of GWAS have been conducted in European beech (Pfenninger et al. 2021; Müller et al. 2024; Lazic et al. 2024), and even less GWAS used the chromosome‐level assembly of European beech (but see Lazic et al. 2024; Pfenninger et al. 2021). GWAS are also conducted in natural populations in forestry research, for example, in beech (Müller et al. 2024; Pfenninger et al. 2021), but concerns due to insufficient control of false positive observations caused by population structure and environmental factors exist (Sillanpää 2011). When leaf samples for GWAS are taken directly from natural populations, trait measurements are also influenced by the environmental conditions of the sampling years and heterogeneity of sampling sites (Fotelli et al. 2003). For example, measurements of traits such as intrinsic water use efficiency can vary greatly between dry and wet years, so environmental conditions must be considered (Fotelli et al. 2003). Therefore, results should be considered carefully but may point out interesting single‐nucleotide polymorphisms (SNPs) candidates to be validated in future studies. Here, we additionally employ a novel approach that complements GWAS with tests of polygenic selection to evaluate whether traits, despite lacking significant SNP associations, are under selection but are too polygenic to pass significance thresholds.

Our study comprises 514 individual trees collected in the Postavarul Massif, South‐Eastern Romanian Carpathian Mountains (Carpații de Curbură) from five European beech stands, along an altitudinal gradient which follows a precipitation gradient. The mean annual precipitation was higher at higher altitudes; hence the lower elevation stands are more likely to experience periods of limited soil water availability. The main goal of this study is to identify candidate SNPs for drought stress traits which could be evaluated in subsequent studies and prioritized in future breeding programs. Several drought stress‐related and physiological traits were measured. These traits include stomatal density. Stomata, located in the plant leaf epidermis, are important to control gas exchange and transpiration (Gailing et al. 2008; Bertolino et al. 2019; Chen et al. 2022). Stomatal characteristics, such as stomatal density and control, are important for adjusting photosynthesis to changes in CO_2_ levels, temperature, photosynthetically active radiation, and water availability (Gailing et al. 2008; Bertolino et al. 2019; Chen et al. 2022). Carbon isotope discrimination (δ^13^C) in leaf dry matter as a proxy for water use efficiency (Fotelli et al. 2003; Querejeta et al. 2003) was also measured as a drought stress‐related trait. Stomatal closure due to reduced soil water availability and altered plant water status reduces the concentration of CO_2_ in the stomatal cavities, and the fixation of the heavier ^13^C isotope increases compared to ^12^C (Fotelli et al. 2003). Consequently, plant water potential can be related to δ^13^C (Fotelli et al. 2003). Furthermore, leaf nitrogen and carbon contents were measured, and the C/N ratio was calculated from these measurements. Leaf nitrogen content and the C/N ratio are impacted by complex physiological mechanisms (Gessler et al. 2017). At the start of a drought event, nitrogen uptake decreases (Gessler et al. 2017). Drought stress can lead to short‐term depletion of leaf nitrogen and long‐term depletion of leaf carbon (Gessler et al. 2017; Ruehr et al. 2019). The impact on the C/N ratio depends on the nutrient availability before the drought stress event; reduced nutrient availability at the beginning of drought stress will result in a higher C/N ratio during drought (Gessler et al. 2017). The heritability of C/N ratio, leaf N and δ^13^C varies based on our literature review from 0.21 to 0.72 in different oak species (McKown et al. 2019; Alexandre et al. 2020).

These drought stress traits, which comprise stomatal density, δ^13^C, C/N ratio, and leaf nitrogen content, were used in previous studies on other plant species (Bhaskara et al. 2022; Li et al. 2023; De La Torre et al. 2022). In comparison to other studies, the different beech stands in this study were not exposed to an artificial drought stress regime, but historic climate data from the stand locations showed big differences in precipitation and temperature along the altitudinal gradient at the different sampling sites.

The relationship of minor allele frequencies (MAF) observed at GWAS candidate loci, altitudinal gradient, and population mean values of traits of interest was further investigated. Nearby annotated genes were identified through a literature review to evaluate their putative roles in local adaptation. However, if the traits are too polygenic to identify SNP candidates, $\hat{G}$ can provide information on whether the traits have a genetic basis and have been subject to polygenic selection. It is assumed that drought stress response has a polygenic basis (De La Torre et al. 2022). But when drought stress is dissected into different drought stress‐related traits (e.g., stomatal conductance, water use efficiency), some appear not to be highly polygenic, so that candidate SNPs were identified in previous studies (e.g., McKown et al. 2019). When the polygenic test $\hat{G}$ is successful, the previously estimated marker effects could also be used for genomic prediction. To our knowledge, $\hat{G}$ has not been applied to forest tree species yet, but it can provide insights into the adaptive potential and genetic landscape that would remain undiscovered with GWAS.

## 2 Material and Methods

### 2.1 Genotypes and Phenotypes

A data set of 514 individual trees was collected from five beech stands in the South‐Eastern Romanian Carpathians (Carpații de Curbură) along an altitudinal gradient associated with changes in precipitation and temperature (Grigoriadou‐Zormpa et al. 2025; Tost et al. 2025). The exact geographical locations and more details about the stands are reported by Grigoriadou‐Zormpa et al. (2025). The beech stands are referred to as Lempes, Tampa, Solomon, Lupului, and Ruia. In Ruia, 110, in Lupului and Lempes 101, in Solomon 102 and in Tampa 99 trees were sampled. Lempes was the lowest‐elevation site at 550–600 m, with the lowest mean annual precipitation of 712 mm, followed by Tampa at 650–700 m with 791 mm, Solomon at 800–900 m with 855 mm, and Lupului at 1000–1150 m with 957 mm mean annual precipitation. Ruia was the highest location at 1300–1450 m with the highest mean annual precipitation of 1023 mm. The tree stands are naturally regenerating, and adult trees are 100–160 years old (Grigoriadou‐Zormpa et al. 2025). Grigoriadou‐Zormpa et al. (2025) analyzed the population structure of the stands and observed a weak population genetic structure. More details about the genetic structure of the population are provided in the previous publication of Grigoriadou‐Zormpa et al. (2025).

The phenotypic variables comprised four traits: leaf nitrogen and C/N ratio in the leaves, carbon isotope composition (δ^13^C) in leaf dry mass, and stomatal density. In 2020, the leaves were sampled from October 15 to October 28; in 2021, from August 16 to August 22; and in 2022, from August 8 to August 13. All traits were measured from leaves collected in 2020, 2021, and 2022, except for stomatal density, which was measured only from leaves collected in 2020 due to the availability of leaf material. In each year, samples were collected from the same set of individual trees. Consequently, the genomic regions identified for this trait might reflect effects specific to that particular year. Stomatal density was measured by counting the stomata within a fixed area of 0.09375 mm^2^ (Yücedağ et al. 2021) on the abaxial side of one dried sun‐exposed leaf per tree sampled in 2020. The stomata were counted based on a stomatal imprint created with fingernail polish and tape from dried leaves (Yücedağ et al. 2021). Carbon isotope composition (δ^13^C) in leaf dry mass, leaf nitrogen and carbon content were measured in 2020, 2021, and 2022. To evaluate changes in δ^13^C in leaf dry mass, leaf nitrogen and carbon content, homogenized plant material was analyzed by the Center for Stable Isotope Research and Analysis at the University of Goettingen by elemental analysis combined with isotope ratio mass spectrometer measurements (Werner et al. 1999). We calculated the C/N ratio based on the molar content (mmol g^−1^) and molar weights of carbon and nitrogen. Stomatal closure due to reduced soil water availability and altered plant water status reduces the concentration of CO_2_ in the stomatal cavities and the fixation of the heavier ^13^C isotope increases compared to ^12^C (Fotelli et al. 2003). Consequently, plant water potential can be related to δ^13^C (Fotelli et al. 2003). δ^13^C is also influenced by changes in CO_2_ levels and photosynthetically active radiation, so this trait shows strong variability across the growing season (Fotelli et al. 2003).

We removed extreme phenotypic outliers. The outliers and distribution of trait measurements are shown in File S1. Additionally, we examined the correlations of the traits with each other, which are shown in File S2. The correlations of the traits measured in different years are shown in File S3. Correlations were analyzed using R version 4.3.1 (R Core Team 2025).

Individual trees were sequenced using single‐primer enrichment technology (SPET) (Scaglione et al. 2019). This sequencing method targets SNPs located within and close to genes 500 bp downstream of the designed probe (Scaglione et al. 2019). The target regions were determined by previous whole‐genome sequencing of a subset of 96 individual trees by IGATech (IGA Technology Services Srl, Udine, Italy n.d.). In total, 100,000 high‐quality target regions were used; these target regions were identified because they are close to genes (IGA Technology Services Srl, Udine, Italy n.d.). Reads were aligned to the *F. sylvatica* reference genome version 2 (Mishra et al. 2021) by using BWA‐MEM v0.7.17 (Li and Durbin 2009). Only aligned reads with a mapping quality ≥ 10 were kept, and duplicated reads were removed. Variant calling was performed, along with filtering for the minimum read count of an allele in a sample, using Freebayes v1.3.6 (Garrison and Marth 2012; Grigoriadou‐Zormpa et al. 2025). Normalization and filtering for read depth > 6 and < 257 were performed using bcftools (Danecek et al. 2011; Grigoriadou‐Zormpa et al. 2025). After this, a total of 838,522 bi‐allelic SNP markers were kept. The data set was filtered by PLINK for minor allele frequency (MAF) > 0.194% (which results in at least two observations at a marker), missingness of ≤ 10% at each SNP marker, and for SNP markers without chromosome annotations. The final data set contained 610,678 SNP markers and 514 individuals used for GWAS. Not for all genotyped individual trees were phenotypic measurements available. For stomatal density, 314 individual trees were phenotyped. Four hundred forty individual trees were phenotyped for δ^13^C in 2020, 457 in 2021, and 454 in 2022. For leaf nitrogen content measurements, 451 individual trees were phenotyped in 2020, 457 in 2021, and 450 in 2022. For the C/N ratio, 440 individual trees were phenotyped in 2020, 454 in 2021, and 450 in 2022. A summary of this information can be found in Table S4.

### 2.2 Genome‐Wide Association Study

To test for associations between SNPs and traits, we used a linear model in PLINK v.1.90 (Purcell et al. 2007) for stomatal density and δ^13^C, which were tested separately. The multivariate model GEMMA (Zhou and Stephens 2014) was used to test the 3 years of measurements for the C/N ratio together. The same procedure was done for leaf nitrogen. The multivariate model of GEMMA was used based on the suggestion of a previous review. The GWAS results for stomatal density and δ^13^C conducted in PLINK appeared less inflated regarding their QQ plots than the GWAS results for stomatal density conducted in GEMMA (see Figure S11). The year‐to‐year correlations of leaf nitrogen content were 0.45, 0.42, and 0.44, and for the C/N ratio, 0.47, 0.46, and 0.51 (also see Figure S3).

The first two main components of a principal component analysis (PCA) of a set of putatively neutral SNPs, which explained together ~12.81%, were included as covariates to account for population structure. Neutral SNP markers were identified by Grigoriadou‐Zormpa et al. (2025) for population genetic analysis. To identify neutral SNP markers, Grigoriadou‐Zormpa et al. (2025) defined intergenic regions using SnpEff (version 4.3t; Cingolani et al. 2012). Afterwards, the filtering based on LD and missingness < 10% was done, which resulted in a data set of 154,437 putatively neutral SNPs. The results from the PCA based on the neutral markers, conducted with the R package “SNPrelate” (Zheng et al. 2012), are available as Figure S5. Only the first two PCs were included to account for population structure, even though they explain only 12.81% of the total variance of neutral markers; the remaining PCs separate the populations in a similar manner (see Figure S5). To account for environmental heterogeneity during sampling in 2020, 2021, and 2022, the first principal coordinate based on precipitation and temperature during the main growing season from April to August (Figure S6 and Table S7) in the year of sampling was included in the GWAS for δ^13^C (Figure S8). This was done because δ^13^C varies strongly across the growing season (Fotelli et al. 2003). Temperature and precipitation data were downloaded from Copernicus Climate Data Store (CDS 2024) for 2020, 2021, and 2022 (available in Figure S6, Table S7, and Figure S8). The principal coordinate analysis (PCoA) was performed on monthly temperature and precipitation measured from April until August for the years separately using the *cmdscale()* R function in the R version 4.3.1 (R Core Team 2025). The idea was to account for variation in precipitation and temperature of the different locations related to drought stress, which may have been experienced by the stands during their development.

Furthermore, highly related individuals with a relatedness coefficient ≥ 0.5 were removed (Yang et al. 2011), which is also shown in Files S9 and S10. For leaf nitrogen content and C/N ratio, we used multivariate linear mixed GWAS models to account for environmental heterogeneity, including the genomic relationship matrix (Yang et al. 2011), using the GEMMA software (Zhou and Stephens 2014) and the first two principal components from the PCA based on neutral markers. For the traits leaf nitrogen content and C/N ratio, these models performed better. δ^13^C measured in the different years was analyzed separately and with PLINK since it was not possible to include the environmental covariates along with the genomic relationship matrix due to their correlation with each other. The GWAS for stomatal density was analyzed with PLINK, while accounting for population confounding and the altitudinal gradient by including the first two principal components from the PCA based on neutral markers. QQ plots are shown in File S11. All stands grow on rendzinas except for the lowest‐located population, which grows on luvisol. We did not account for different soil conditions in this study, but for future studies, we would also recommend testing for differences due to soil properties, which may influence drought stress levels, or selecting populations growing on similar soils.

The significance thresholds were derived based on a permutation test using the same set of SNP markers (Purcell et al. 2007). The permutation test was conducted in adaptive permutation mode, which can terminate before completing all possible iterations when there are no further changes (Purcell et al. 2007). This adaptive permutation mode was run with a minimal of 10 and a maximal of 1000,000 permutations. The level at which no markers were identified as significant in our permutation test was declared the significance threshold, which in our case was *p* ≤ 0.000001 for all traits.

### 2.3 Calculation of *F*_*ST*_ *Sum*

Selection signature mapping was based on the *F*_ST_ statistic. *F*_ST_ was calculated as $F_{\mathrm{ST}}=\frac{s^{2}}{\mu \left(p\right)*\mu \left(1-p\right)+\frac{s^{2}}{r}},$ where *μ*(*p*) is the mean allele frequency among the stands, *s* ^2^ is the sample variance of the allele frequency among stands, and *r* is the number of analyzed stands at a marker (Weir and Cockerham 1984). The *F*_ST_ statistics were calculated among all stands comprising Ruia, Lupului, Solomon, Tampa, and Lempes at each marker.

### 2.4 Gene Variant Annotation and Functional Categories

SnpEff (version 4.3t, Cingolani et al. 2012) was used for gene variant annotations. An annotation database was built from the *F. sylvatica* reference genome (Mishra et al. 2021), which was also used in this study for gene variant annotation. SnpEff uses this database and the VCF file from this study to annotate gene variants, their effects and predicted impacts. The effects include missense variants, stop‐codon variants, frameshift variants, splice‐site variants, and several others. A missense variant causes a codon change that results in a different amino acid. We created a pairwise LD heat map using the R package “LDheatmap” for regions containing SNP markers associated with the traits and interesting gene annotations (Shin et al. 2006). We derived information about functional categories of the genes from the KEGG database (Kanehisa et al. 2025; Kanehisa 2019; Kanehisa and Goto 2000).

### 2.5 Haplotype Analysis and LD Block Determination

The data were phased using fastPHASE version 1.4.8 (Scheet and Stephens 2006). We phased the genomic data of the regions significantly under selection with 10 iterations of the expectation–maximization (EM) algorithm (Scheet and Stephens 2006). The phased haplotypes were used to create a pairwise LD heatmap with the R package “LDheatmap” for the region of interest (Shin et al. 2006).

### 2.6 Ghat as a Test for Polygenic Selection

**FIGURE 1:**
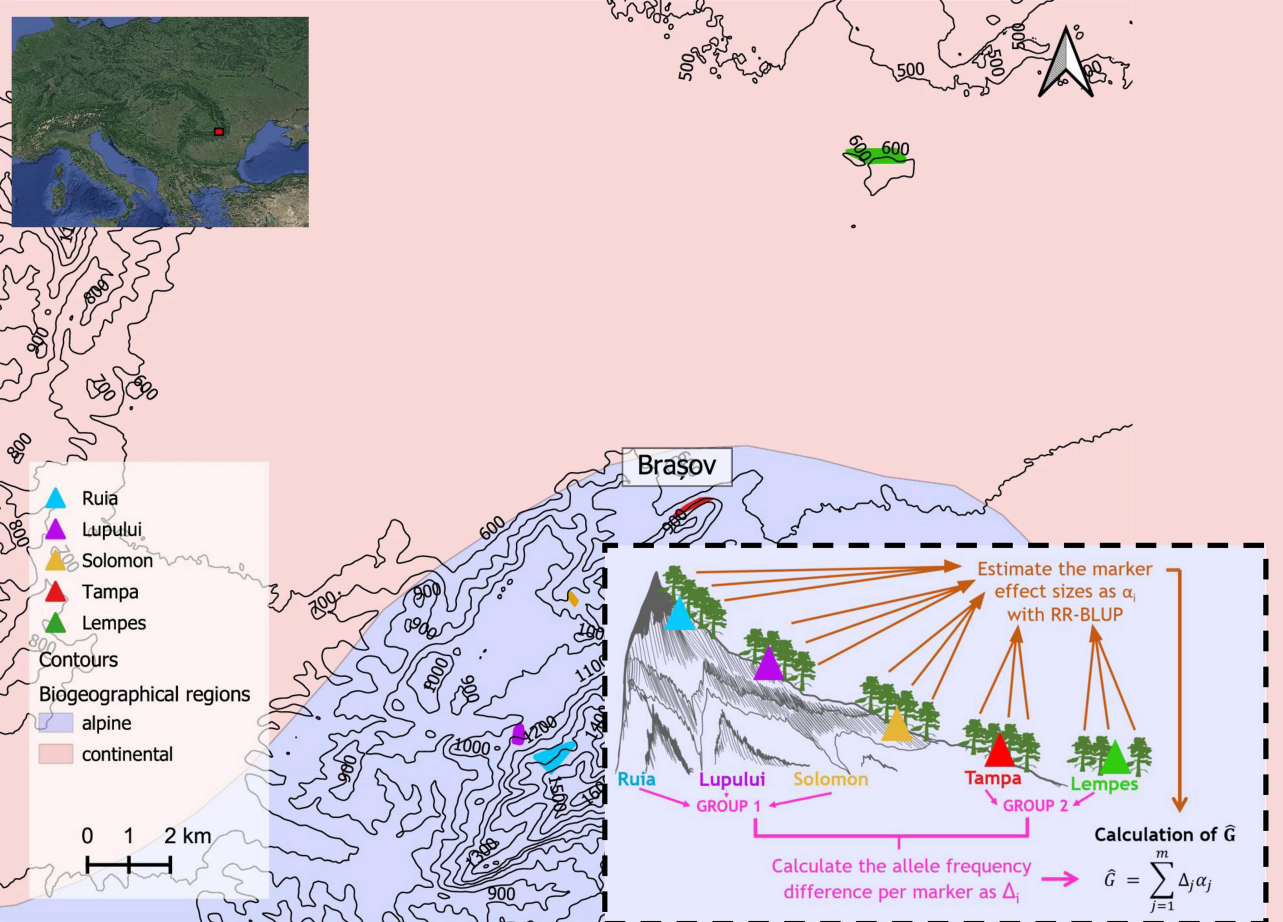
Map of Postavarul Massif, South‐eastern Carpathians (Carpații de Curbură) forests covering a transect from 500 to 1500 m (a.s.l.) (Grigoriadou‐Zormpa et al. 2025) with simplified scheme of the location of the beech stands along the altitudinal gradient and the calculation of $\hat{G}$ by evaluating the marker effect sizes and allele frequency differences between groups of different environmental conditions across the entire genome.

Groups of stands growing under different environmental conditions were treated as groups that faced different selective regimes to implement $\hat{G}$ (Figure 1). The groups were formed based on altitude and the amount of precipitation (Figure 1). Group 1 included the stands Ruia and Lupului, which were located at altitudes > 1000 m and with a mean annual precipitation > 990 mm (Figure 1). Group 2 included the stands Tampa and Lempes, which were located at altitudes < 800 m and with a mean annual precipitation < 800 mm (Figure 1). Solomon was removed from the $\hat{G}$ analysis, to increase the difference in environmental conditions between the two groups (Figure 1). The marker effect estimates were calculated, by using the random regression best linear unbiased prediction (rrBLUP) model available in the “rrBLUP” R package (Endelman 2011).

Filtering for minor allele frequency (MAF) and missingness < 0.05 was done before missing observations were imputed based on the mean allele frequency of the stands observed at each marker in R version 4.3.1 (R Core Team 2025). When $\hat{G}$ was calculated for δ^13^C, the weather data of the different years of sampling (see Figure S6 and Table S7) was included in the model to estimate the marker effects. $\hat{G}$ was calculated with the “Ghat” R package (Mahmoud et al. 2023). The number of permutations was set to 1000. The number of effective markers was first calculated using the “ld_decay” function from the Ghat package (Mahmoud et al. 2023). To evaluate its impact on $\hat{G}$, we additionally tested 100 randomly generated traits. These 100 random traits were generated by simulating a normal distribution with a mean of 25.93 and a standard deviation of 3.67 (mean and standard deviation of the trait leaf nitrogen content), then the traits were randomly associated with the individual trees. In the test runs, we also tested whether these random traits are identified as being under significant selection. When the random traits were identified as under significant selection, they were considered false‐positive observations. Based on the 100 random traits, the false‐positive rate was calculated and used to evaluate the number of effective markers. Later, in the actual $\hat{G}$ test, we tested the four different traits stomatal density, δ^13^C, leaf nitrogen content and C/N ratio to identify signals of polygenic selection.

### 2.7 Cross‐Validation of Marker Effect Sizes

To evaluate the marker effect estimates used in the $\hat{G}$ test, we conducted a cross‐validation. For cross‐validation, the data set was randomly partitioned into five sets for five‐fold cross‐validation with 100 replications (Endelman 2011). In the cross‐validation, phenotypic values for each trait in the test set were predicted from marker effect estimates calculated on the training set (Endelman 2011). The marker effect estimates were calculated with rrBLUP, using the same method as for the $\hat{G}$ test (Endelman 2011). The predictive ability was calculated as correlation of predicted trait estimates and observed trait measurements for the different traits of the testing set (Endelman 2011). Additionally, another cross‐validation was conducted in which one stand was left out and the remaining stands were used for prediction. With this cross‐validation we could assess how well the stands can predict a new stand.

## 3 Results

### 3.1 GWAS Revealed a Peak on Chromosome 10 Associated With Stomatal Density and δ^13^C

**FIGURE 2:**
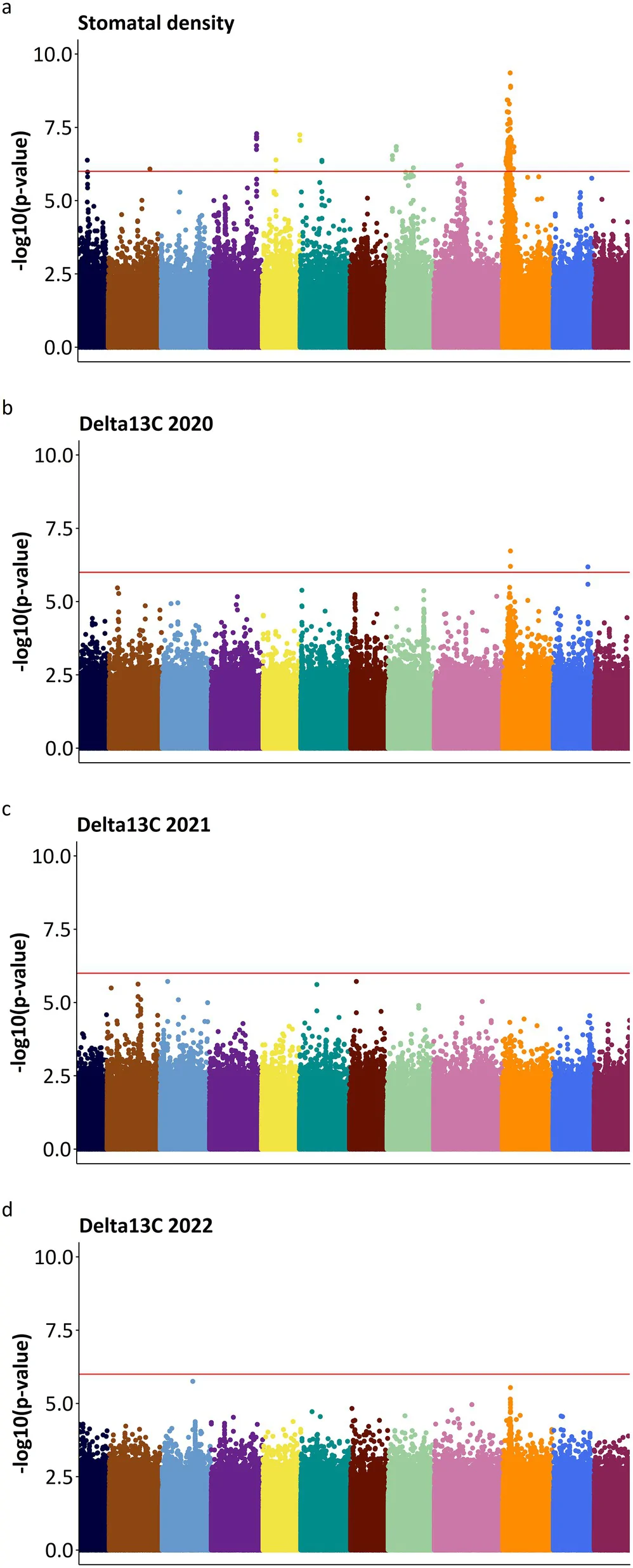
Manhattan plot for stomatal density in 2020 (a), for δ^13^C measured in 2020 (b), 2021 (c), and 2022 (d) with premutation derived significance thresholds of *p* ≤ 0.000001 (red horizontal line) with *p* values plotted as −log_10_(*p*) against the position in bp. In 2020, significant associations for stomatal density and δ^13^C are found at overlapping positions on chromosome 10.

Stomatal density was significantly associated with 88 markers, of which 72 are located on chromosome 10 (Figure 2). The remaining markers are located on chromosomes 1, 2, 4, 5, 6, 8, and 9, with one to five markers on each chromosome (Figure 2).

**FIGURE 3:**
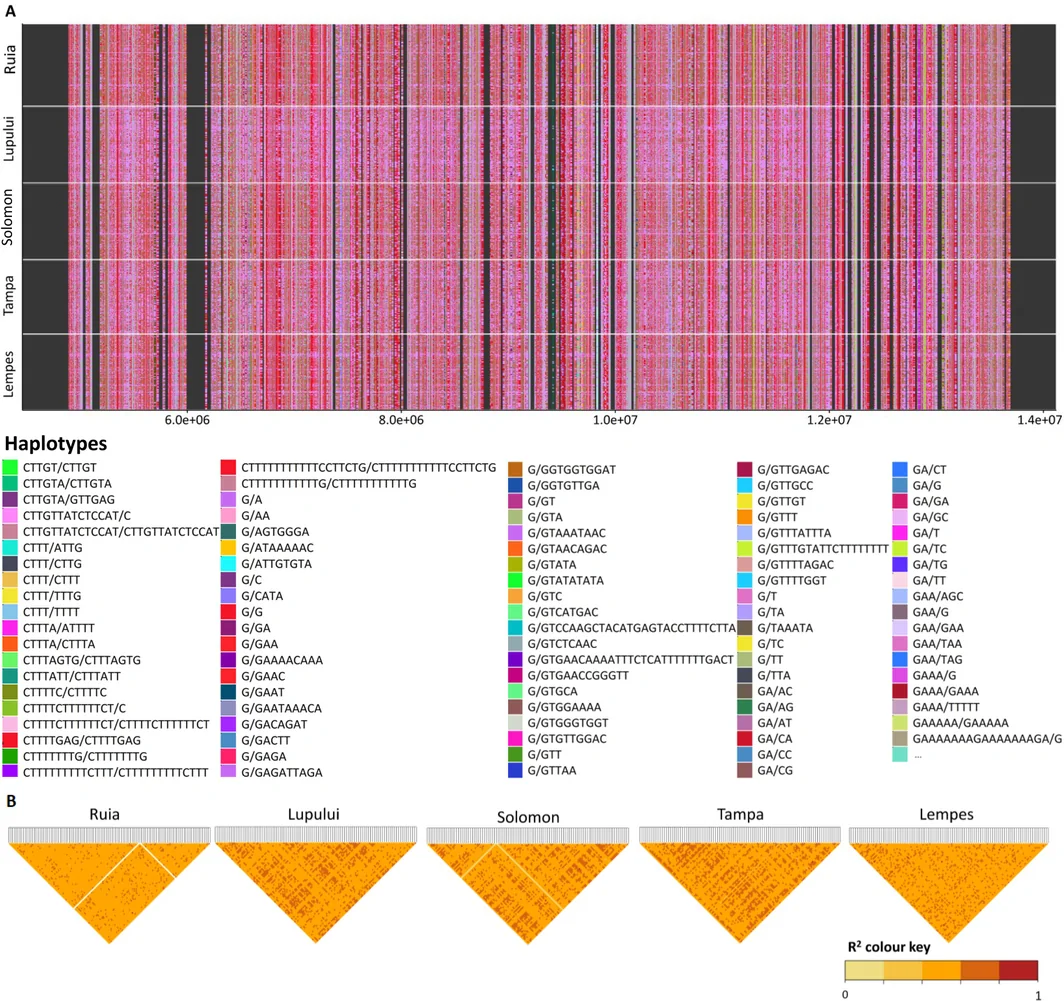
Region on chromosome 10 from ~5.48 to ~13.67 Mb and the observed haplotypes (A) and pairwise LD heat map for the different stands (B).

In total, 72 of these markers are located between positions ~5.48 to ~13.67 Mb on chromosome 10 and appear as one wide peak. Figure 3 displays the observed haplotypes across this entire region and the pairwise LD heat map within the different stands. All 72 markers showed increased linkage disequilibrium (*R* ^2^ = 0.5–0.6) in stands Solomon, Tampa, and Lupului. Observed haplotypes across the entire region differ especially between Ruia and Lupului, and between Tampa and Lempes (Figure 3A). Pairwise LD is increased in Lupului, Solomon, and Tampa compared with Ruia (Figure 3B).

A similar peak on chromosome 10 appears in the GWAS for δ^13^C measured in 2020 and 2022 (Figure 2). But this peak is smaller and contains almost no significant SNP markers (Figure 2). Only for δ^13^C measured in 2020, three SNP markers exceeded the significance threshold (Figure 2). No significant SNPs were associated with δ^13^C measured in 2021 and 2022 (Figure 2).

Gene annotations were found in close proximity to 26 of the 72 SNP markers. Table 1 presents the most interesting candidate SNPs for stomatal density, focusing on candidate SNPs within 20 kb of gene annotations. The complete list of significant SNPs for stomatal density and δ^13^C, measured in 2020, is provided in Table S12. The SNP markers significantly associated with δ^13^C measured in 2020 were not located within 20 kb of impactful parts of coding regions (Table S12).

**TABLE 1:** List of candidate SNPs with gene annotations associated with stomatal density with permutation derived significance levels of *p* ≤ 0.000001 and their annotated gene variants, the underlying genes with their locations, functions and predicted impact (HIGH, MOD (moderate), and LOW) based on SnpEff (Cingolani et al. 2012). The predicted impact is calculated with the SnpEff software by analyzing the effect of the variant on the coding region of the annotated gene (Cingolani et al. 2012). For example, stop gains are assumed to be of high impact, whereas missense variants leading to different amino acids are assumed to be of moderate impact (Cingolani et al. 2012). All annotations were obtained from the NCBI database (2025).

Chr	Position of SNP	Distance to gene variant in kb	Gene variants		Impacts	Genes
10	7198804	~13.6 to 13.8	Bhaga_10.g867	7 missense variants	MOD	XP_030956796.1: capsanthin/capsorubin synthase, chromoplastic‐like
	7211779	−18.4	Bhaga_10.g873	Variant hits 5'UTR region with premature start codon gain variant	LOW	XP_030956765.1: mRNA‐decapping enzyme‐like protein
	7555433	3.1	Bhaga_10.g917	stop codon	HIGH	XP_030964327.1: aluminum‐activated malate transporter 2‐like isoform X1. The *aluminum‐activated malate transporter (ALMT)* gene family which is involved in tolerance to Al^3+^ and regulation of opening and closing of stomata in plants (Dabravolski and Isayenkov 2023).
	7555433	~3.2 to 3.3		9 missense variants	MOD	
	7785927	~15.9 to 16.1	Bhaga_10.g957	30 missense variants	MOD	XP_030940718.1: transcription factor MYB35‐like isoform X2. MYB related transcription factors are involved in chloroplast biogenesis and are targeting genes for carbon fixation (Frangedakis et al. 2024).
	7785978					
	7787468					
	7820271					
	7820389					
	11903957	14.3	Bhaga_10.g1460	3 missense variants	MOD	XP_030939393.1: ATP‐dependent Clp protease adapter protein *ClpS1*, chloroplastic‐like. *ClpS1* forms together with *ClpF* an important unit in the chloroplasts which is involved in substrate recognition and delivery (Nishimura et al. 2015).
	11903957	~‐11.5 to −11.7	Bhaga_10.g1462	5 missense variants	MOD	XP_030938815.1: protein ASPARTIC PROTEASE IN GUARD CELL 1. The overexpression of *ASPG1* was observed to lead to an increase of abscisic acid (ABA) sensitivity in stomatal guard cells and leads to reduced water loss in transgenic *Arabidopsis* plants (Yao et al. 2012).
	13435289	located within the coding region	Bhaga_10.g1651	Synonymous gene variant		XP_023902123.1: protein BONZAI1. *BONZAI* genes control global osmotic stress responses, ABA accumulation and stomata regulation in plants (Chen et al. 2020).
		~4.8		1 splice region variant	LOW	
		~‐14.6	Bhaga_10.g1652	1 missense variant	MOD	VVA19309.1: uncharacterized LOC117629398
	13677756	~‐11.8	Bhaga_10.g1674	22 missense variants	MOD	XP_034215671.1: poly(A) polymerase I‐like isoform X1
	13677816					

The gene variant Bhaga_10.g917 corresponds to the underlying gene *aluminum‐activated malate transporter 2‐like* (*ALMT2*) (NCBI 2025). A stop codon of the gene *ALMT2* is located ~3.1 kb upstream from the marker found on chromosome 10 at ~7.56 Mb that was associated with stomatal density (see Figure S13). The stop codon is located in the center of the gene (Figure S13). Furthermore, nine missense variants of the gene's coding region are located ~3.2 to 3.3 kb upstream from the marker. According to the pairwise LD (linkage disequilibrium) heat map analysis, we observed strong LD around the marker and this gene (see Figure S13).

We found five markers on chromosome 10 at ~7.78 to ~7.82 Mb, which were ~16 kb upstream from 30 missense variants located within the coding region of *transcription factor MYB35‐like* with a moderate impact on its expression (Table 1; see also Figure S14). Our pairwise LD heat map, available as Figure S14, shows that the significant marker is in almost complete LD (~1) with the coding region of this gene.

The marker on chromosome 10 at ~11.9 Mb is located in the intergenic region between *Bhaga_10.g1460* (*ClpS1*) and *Bhaga_10.g1462* (*ASPG1*) (Table 1), but the pairwise LD is only increased in some parts along this region (see also Figure S15). It is also located ~12 kb downstream from five missense variants of *Bhaga_10.g1462* (*ASPG1*) (see also Figure S15).

**FIGURE 4:**
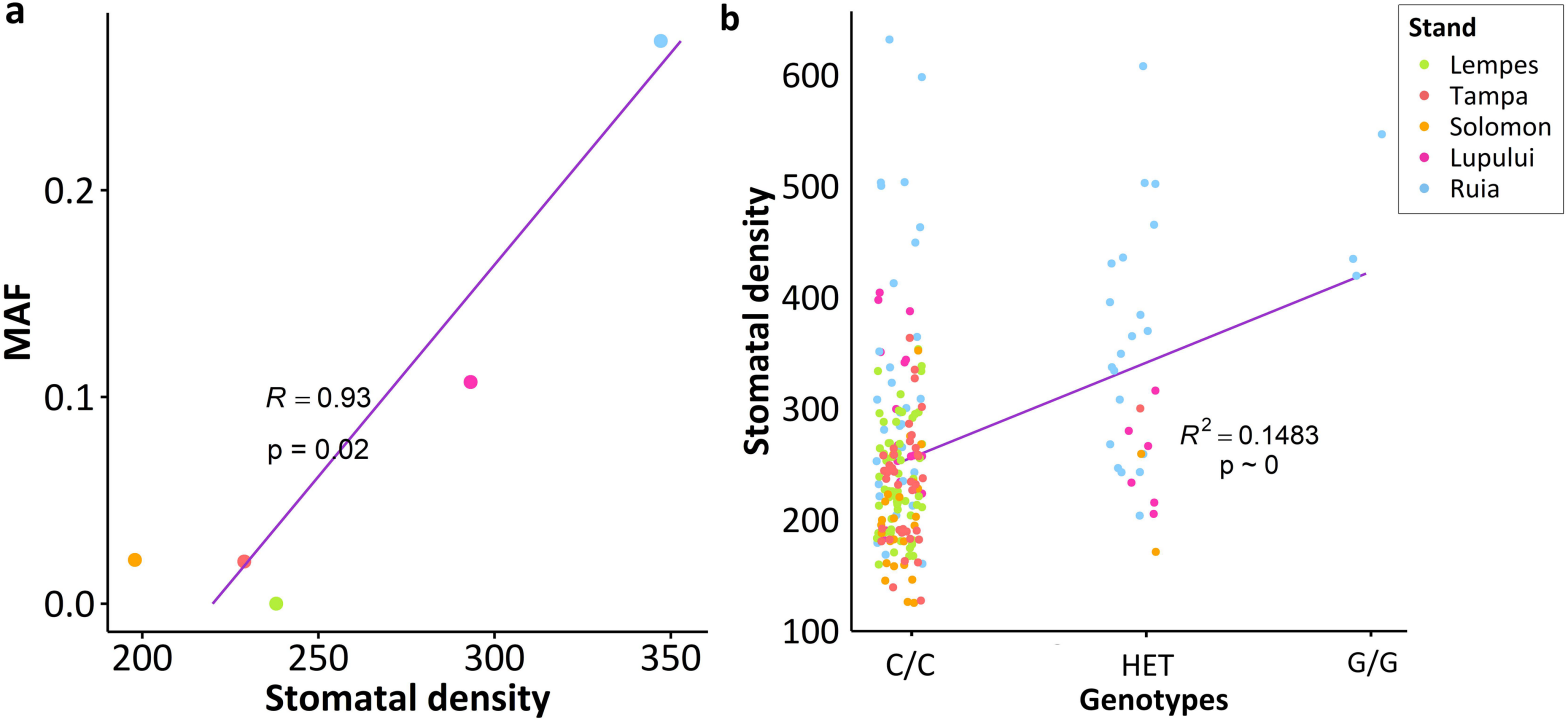
(a) Correlation between mean stomatal density per population and the minor allele frequency (MAF) of the significant marker (*p* ≤ 0.000001) on chromosome 10 at ~13.435 Mb with a correlation coefficient of 0.932 (*p*‐value = 0.021) and (b) Linear regression of marker genotypes against stomatal density with a coefficient of determination *R* ^2^ of 0.1483 (*p* = 0.0001258). This marker was annotated with gene variant *Bhaga_10.g1651*, with the underlying gene *XP_023902123.1*, also described as *BONZAI1*.

The significant marker on chromosome 10 at ~13.44 Mb is located within the coding region of *Bhaga_10.g1651* (*BONZAI1*) (synonymous gene variant) and ~4.8 kb downstream from this marker is a splice region variant of this gene (Table 1; see Figure S16). The pairwise LD is only increased in a segment of this region (Figure S16). The gene variant *Bhaga_10.g1652* is located ~14 kb downstream of this marker, but no gene description was found for it (see Figure S16). One missense variant was found in the coding region of *Bhaga_10.g1652*, which is ~14.6 kb downstream of the significant marker on chromosome 10 at ~13.44 Mb (Figure S16). The MAF at this marker increases with increasing stomatal density, which follows the altitudinal gradient associated with precipitation and temperature (Figure 4). For all other markers on chromosome 10 from ~5.48 to 13.67 Mb, we observed the same trend (see Table S18 and Figure S19). In all cases, it is indicated that the MAF is related to the mean stomatal density within populations (Table S18 and Figure S19).

Another gene, *poly(A) polymerase I‐like* (NCBI 2025), was discovered to be involved in regulation processes. A potential role in stomatal density could not be directly inferred from the literature, and further studies are needed (for detailed information, see Table S17).

**FIGURE 5:**
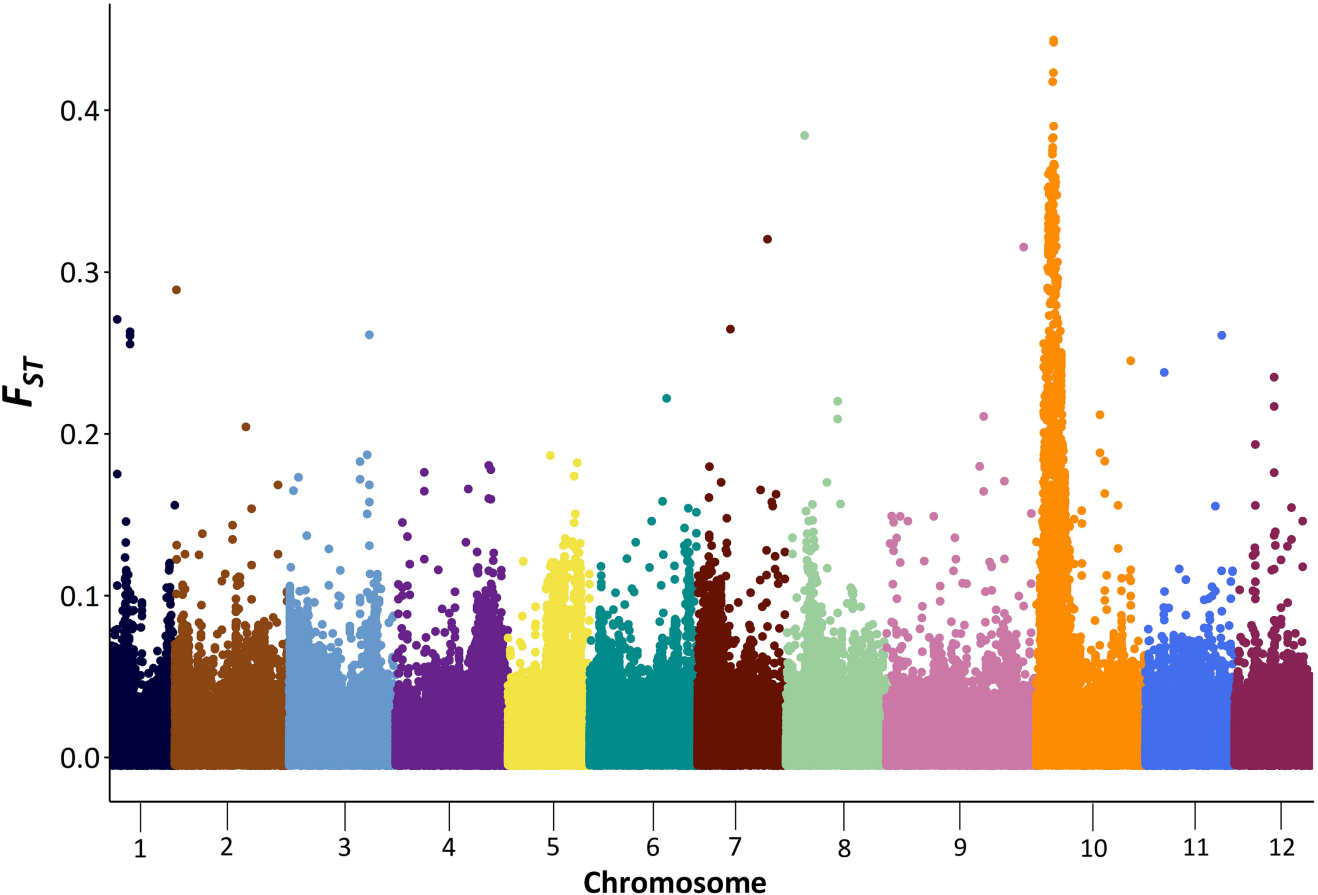
Genome‐wide *F*_ST_ values based on European beech ( *Fagus sylvatica* L.) comprising ~100 adult beech trees per stand in five locations sampled in the South‐Eastern Romanian Carpathians along an altitudinal gradient associated with precipitation and temperature.

Further evidence supporting that this region on chromosome 10 is involved in local adaptation is provided in Figure 5. *F*_ST_‐based selection signature mapping revealed a peak on chromosome 10 from ~4.512 to ~47.742 Mb (Figure 5). This peak is partly overlapping with the peak containing marker associations from the GWAS for stomatal density from ~5.48 to ~13.67 Mb on chromosome 10.

### 3.2 Associations of SNP Markers With Leaf Nitrogen and Carbon Traits Differed Across Years of Sampling

**FIGURE 6:**
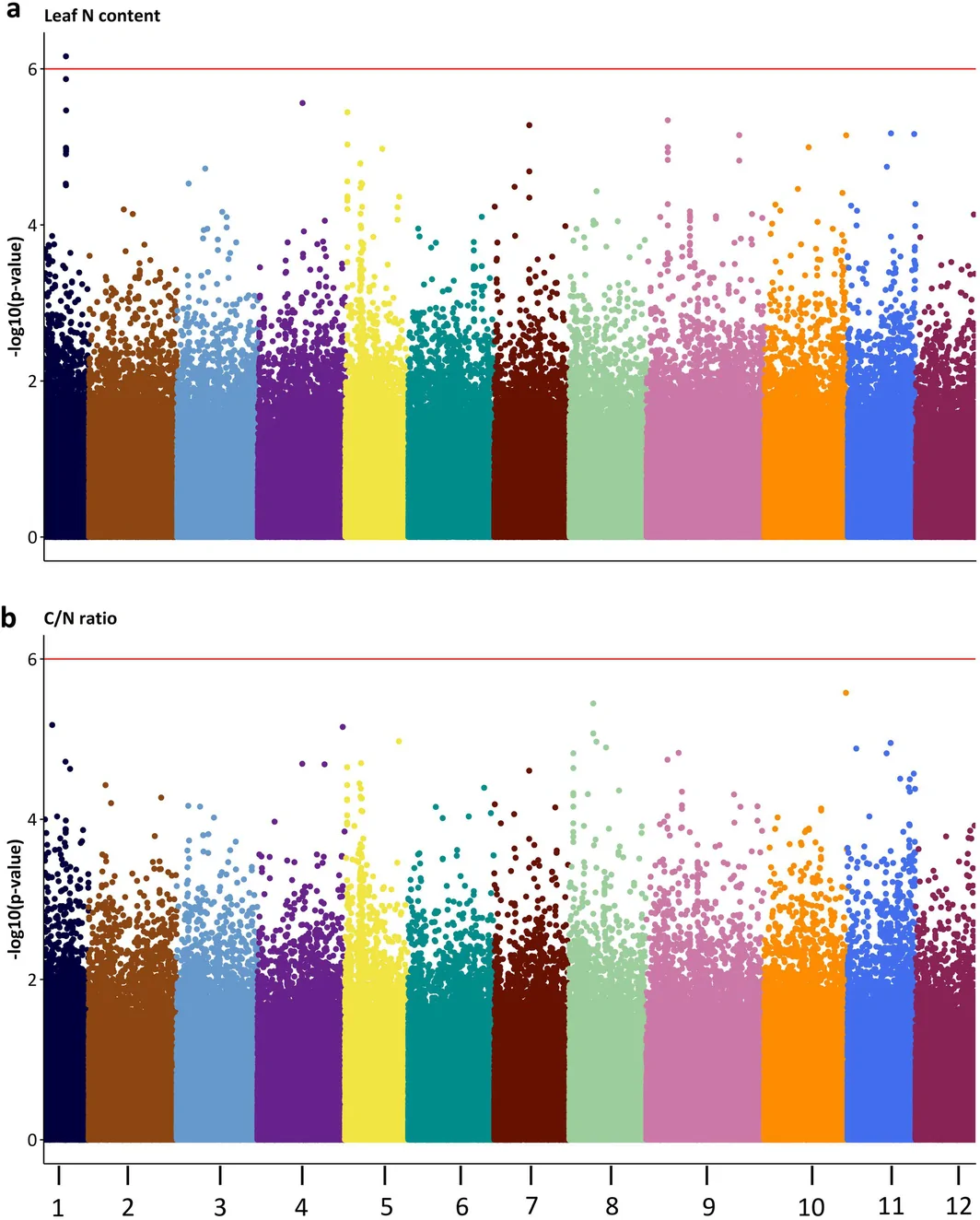
Manhattan plot for leaf nitrogen content (a) and C/N ratio (b), with permutation derived significance thresholds of *p* ≤ 0.000001 (red horizontal line) with *p* values plotted as −log_10_(*p*) against the position in bp. Leaf nitrogen content and C/N ratio measured in 2020, 2021, and 2022 were each analyzed in a multi‐GWAS using GEMMA.

Figure 6 displays the Manhattan plots for the other traits with significant associations, namely leaf nitrogen content and C/N ratio measured in 2020, 2021, and 2022 using a multi‐GWAS across years. For C/N, no significant hit was found, while for leaf nitrogen content, one significant association was detected (Figure 6a). Gene annotations of genes < 20 kb away from this SNP candidate are shown in Table 2.

**TABLE 2:** List of candidate SNPs associated with leaf nitrogen content (NC) with permutation derived significance levels of *p* ≤ 0.000001 and their annotated gene variants, the underlying genes with their locations, functions and predicted impact (HIGH, MOD (moderate), and LOW) based on SnpEff (Cingolani et al. 2012). Annotations were obtained from the NCBI database (2025) and Uniprot (2025).

Traits	Chr	Position of SNP	Distance to gene variant in kb	Annotated gene variants		Impacts	Genes
NC	1	38020777	~9 kb	Bhaga_1.g3587	3 splice region variants	LOW	Protein sequence of the protein PIR isoform X3 studied in *Quercus lobata* L
			4.5 kb		3 missense variants	MOD	
			−0.284		splice region variant	LOW	
			−0.195		missense variant	MOD	
			−0.172		stop codon	HIGH	

For leaf nitrogen content, we did not observe a consistent trend of MAF for the significant markers (see Table S20 and Figure S21). Figure S21 shows the correlations for MAF and leaf nitrogen content in 2020, 2021, and 2022, which were not significant (*p*‐values 0.84, 0.28, 0.33, respectively).

### 3.3 Ghat Indicates Signals of Positive Polygenic Selection for Leaf δ^13^C

To evaluate the calibration of $\hat{G}$, we tested 100 randomly generated traits. The calculated number of effective markers estimated based on LD decay over the entire genome was 838,522, which corresponds to the number of total markers. This resulted in 90 out of our randomly generated traits appearing as under selection by the uncalibrated $\hat{G}$ test. We did a grid search for the effective marker number (ranging from 1000 to 800,000). With 7000 effective markers, we observed a rate of false positive observations < 10% (see File S22). Results from cross‐validation of predictions using marker effect estimates from 100 replications are available in File S23.

We calculated $\hat{G}$ between the high precipitation (Ruia and Solomon) and low precipitation group (Tampa and Lempes) and observed positive selection with a correlation coefficient between allele effect estimates and allele frequency change of 0.1017 (*p* ~ 0) and 0.0962 (*p* ~ 0) for δ^13^C in 2020 and 2021 (see Table S24). Based on this observation, we can deduce that δ^13^C decreased with increasing precipitation along the altitudinal gradient (see also Figure S1; panel L). $\hat{G}$ calculated based on δ^13^C measured in 2022 was not significant (Table S24). Soil water availability and plant water status can be related to δ^13^C, because an increase in δ^13^C describes the reduced concentration of CO_2_ in the stomatal cavities under closed stomata to improve the plant water potential (Fotelli et al. 2003). Under these circumstances, water use efficiency is usually increased (Fotelli et al. 2003). For the C/N ratio, no signals of polygenic selection were observed (Table S24). Negative selection with a correlation coefficient between allele effect estimates and allele frequency change of −0.0383 (*p* ~ 0.0014) was observed for leaf nitrogen measured in 2021, see Table S24.

## 4 Discussion

### 4.1 Annotations of Genes in LD With SNPs Associated With Stomatal Density Relate to Regulation of Plant Water Status and Stomatal Characteristics

Close to SNP markers significantly associated with stomatal density, five genes were identified with direct influence on stomata regulation, chloroplast metabolism, and plastid maintenance (Gou et al. 2015; Nishimura et al. 2015; Shen et al. 2018; Dabravolski and Isayenkov 2023; Wang et al. 2024; Frangedakis et al. 2024) in *Arabidopsis thaliana* L. (see Table 1). These genes with a direct influence on stomatal characteristics comprise *aluminum‐activated malate transporter 2‐like* (Dabravolski and Isayenkov 2023), *transcription factor MYB35‐like* (Frangedakis et al. 2024), *ATP‐dependent Clp protease adapter protein ClpS1* (Nishimura et al. 2015), *aspartic protease in guard cell 1* (Shen et al. 2018; Wang et al. 2024), and *BONZAI1* (Gou et al. 2015).

The gene variant *Bhaga_10.g917* corresponds to the underlying gene *aluminum‐activated malate transporter 2‐like (ALMT2)* (NCBI 2025). *Aluminum‐activated malate transporter 2‐like (ALMT2)* belongs to the aluminum‐activated malate transporter (*ALMT*) gene family, which is involved in tolerance to Al^3+^, regulation of opening and closing of stomata in plants (Dabravolski and Isayenkov 2023) and fruit flavor (Palmer et al. 2016). This family can be found in many plant species (Palmer et al. 2016). Li et al. (2023) identified *Aluminum‐activated malate transporter 12 (ALMT12)* in a GWAS and a co‐expression network analysis in *Populus*. *ALMT12* has been reported to be involved in stomatal closure in *Arabidopsis* (Palmer et al. 2016). So far, *ALMT2* has been studied in *Zea mays* L. and observed to be responsible for the transport of organic acids or anions in the xylem (Palmer et al. 2016). But this gene may also have another function in European beech, and could also be involved, similar to *ALMT12*, in stomatal control. At the four missense variants, a hydrophilic amino acid was replaced by a hydrophobic one (see Figure S25). The substitution of a hydrophilic amino acid with a hydrophobic one can increase the stability of the resulting proteins in vivo (Lindman et al. 2010). In two cases, the original amino acid was replaced by a cysteine, which could result in a stable change of protein conformation via the formation of disulfide bridges (Ozaki et al. 1987).

*ALMT12* belongs to the functional category of signaling and cellular processes (see Figure S26) (Kanehisa et al. 2025; Kanehisa 2019; Kanehisa and Goto 2000). It is the only annotated gene from this functional group.

The gene variant *Bhaga_10.g957* corresponds to the underlying gene *transcription factor MYB35‐like* (NCBI 2025). MYB‐related transcription factors like *transcription factor MYB35‐like* control chloroplast biogenesis and target genes for carbon fixation and light harvesting (Frangedakis et al. 2024). Mutants of MYB‐related genes appear bleached and contain small chloroplasts (Frangedakis et al. 2024). MYB‐related transcription factors control chloroplast biogenesis in *Arabidopsis* (Frangedakis et al. 2024) and may also control them in tree species like beech, but to draw conclusions, more studies would be needed. For 25 out of the 30 missense variants, no change in the polar bonds was detected (see Table S25). At three missense variants, a hydrophilic amino acid was replaced by a hydrophobic amino acid (see Table S25). Glutamic acid was replaced by lysine, alanine was replaced by valine, and arginine was replaced by glutamine (Table S25). In the remaining two missense variants, one hydrophobic amino acid was replaced by a hydrophilic amino acid (see Table S25). *MYB35‐like* belongs to the functional category “biological process” which is involved in genetic information processing (Figure S26) (Kanehisa et al. 2025). This assignment makes sense since *MYB35‐like* is a transcription factor which may control chloroplast biogenesis in beech.

One marker on chromosome 10 was located between the two gene variants *Bhaga_10.g1460* and *Bhaga_10.g1462* (Figure S21). For the gene variant *Bhaga_10.g1460*, the annotated gene *ATP‐dependent Clp protease adapter protein ClpS1*, also referred to as *ClpS1*, was found (NCBI 2025). *ClpS1* forms together with *ClpF* an important unit in the chloroplasts which serves as an adaptor and is responsible for substrate recognition and delivery (Nishimura et al. 2015). *ClpS1* seems to be important for chloroplast metabolism in *Arabidopsis* (Nishimura et al. 2015) and may have a similar function in beech. At two missense variants of *Bhaga_10.g1460* (*ClpS1*), Glycine was replaced by Alanine and Isoleucine was replaced by Valine (see Table S25). Based on the KEGG database (Kanehisa et al. 2025), *ClpS1* belongs to the functional category “genetic information processing” (Figure S26).

The gene variant *Bhaga_10.g1462* was annotated as the gene *aspartic protease in guard cell 1*, also known as *ASPG1* (NCBI 2025). Stomata pores are surrounded by guard cells (Negi et al. 2014). *ASPG1* was found to be overexpressed in the guard cells under drought stress in *Arabidopsis* (Yao et al. 2012). Overexpression of the *ASPG1* gene increases abscisic acid (ABA) sensitivity in stomatal guard cells and reduces water loss in transgenic *Arabidopsis* plants (Yao et al. 2012). Shen et al. (2018) also observed that this gene plays a role in seed dormancy, seed longevity, and seed germination in *Arabidopsis*. In general, aspartic proteases are involved in various biological processes like chloroplast metabolism, biotic and abiotic stress responses, and reproductive development (Wang et al. 2024). At three missense variants of *Bhaga_10.g1462* (*ASPG1*), a hydrophilic amino acid was replaced by a hydrophobic amino acid (see Table S25). The *ASPG1* gene belongs to the functional category “metabolism” responsible for chloroplast metabolism, biotic and abiotic stress responses.

For the gene variant *Bhaga_10.g1651*, the gene *BONZAI1* was annotated (NCBI 2025). *BONZAI* genes control global osmotic stress responses, ABA accumulation and stomata regulation in plants (Chen et al. 2020). *BONZAI1* regulates Ca^2+^ signaling under osmotic stress in rice (Chen et al. 2020). In *Arabidopsis*, *BONZAI1* was found to promote stomatal closure and ABA accumulation (Gou et al. 2015). ABA plays an important role in stomatal closure in *Arabidopsis* (Chen et al. 2022) and perennial woody species such as poplar (Popko et al. 2010). For *BONZAI1*, no functional category was found, but this gene may belong to the functional category of metabolism.

Several of the gene variants without gene annotations (Table S12), but also *Bhaga_10.g1674* annotated as the gene *poly(A) polymerase I‐like gene* (Table S17), may be involved in stomatal features, but they need to be analyzed further in subsequent studies. It may also be possible that some of these SNP marker associations are due to hitchhiking, because strong joint selection is acting across the entire region on chromosome 10 from ~5.48 to ~13.67 Mb.

### 4.2 Significant Correlations of Stomatal Density With Allele Frequency Along the Altitudinal Gradient Indicate Local Adaptation

In this study, we observed increasing stomatal density with increasing altitude and precipitation and decreasing temperature (see Figures S18 and S19). Accordingly, Hamanishi et al. (2012) observed in *Populus* that lower stomatal density restricts the number of sites for water loss, thereby reducing transpiration. Li et al. (2023) found that under drought stress, *Populus* trees exhibit lower stomatal densities but increased stomatal length and width, along with improved stomatal control mechanisms. In this study, we observed a correlation of stomatal density with water use efficiency measured as δ^13^C measured in 2020 at 0.23 (*p* ≤ 0.005), in 2021 at 0.18 (*p* ≤ 0.005), and in 2022 at 0.18 (*p* ≤ 0.001) (Figure S2). Overall, we found slightly more genes involved in stomatal control and regulation than in stomatal development. Stomatal density and stomatal conductance are usually positively correlated; in this study, stomatal conductance was not measured (Franks et al. 2009).

We observed very similar pairwise relationships between stomatal density and MAF across all markers on chromosome 10 between ~5.48 to ~13.67 Mb (Figure 4, Files S9 and S10). This indicates joint selection across this entire region, in which at least five candidate genes may directly influence stomatal density. However, the pairwise relationship of MAF and stomatal density is not directly related to the position of the stands along the altitudinal gradient (Figure 4, Files S9 and S10). Solomon and Tampa have slightly lower stomatal density than the lowest elevation population, Lempes. This might be explained by the steeper slopes in Solomon and Tampa than in Lempes or by the soil type (luvisol in Lempes, redzina in all other sites), which may have led to less water being retained over the years in Solomon and Tampa in comparison to Lempes. Redzina soils have higher infiltration but limited water storage capacity and are thus more prone to summer desiccation than luvisols (Alexandrovskiy 2000).

Most GWAS for stomatal density identified several genes or QTL (Quantitative Trait Loci) (Gailing et al. 2008; McKown et al. 2019; Chhetri et al. 2020; Li et al. 2023). Based on our GWAS and a literature review, we can assume stomatal density has oligogenic trait architecture and the identified region on chromosome 10 from ~5.48 to ~13.67 Mb seems to be very important in local adaptation. High peak SNP associations and an oligogenic trait architecture for stomatal density were reported by McKown et al. (2019), Chhetri et al. (2020) in *Populus trichocarpa* Hook and by Li et al. (2023) in *Populus* × *tomentosa* Carrière. A linear regression of the different genotypes observed at the SNP on chromosome 10 at ~7.82 Mb and stomatal density is available in Figure S27. The coefficient of linear regression calculated was considerably high (*R* ^2^ = 0.2353, *p* ~ 0).

All gene variants except *Bhaga_10.g1674* were annotated using other tree species in the family Fagaceae, specifically *Quercus suber* L. and *Quercus lobata* Née (NCBI 2025), thereby increasing the reliability of the annotated gene variant results due to their phylogenetic proximity. In contrast, *Bhaga_10.g1674* was annotated based on *Prunus dulcis* Batsch, which belongs to the family Rosaceae (NCBI 2025), and therefore, the annotation may be less reliable.

### 4.3 GWAS Results for Leaf Nitrogen and Carbon Varied Across Traits and Year of Sampling

In total only one SNP marker was significantly associated with leaf nitrogen content. No SNP markers were significantly associated with the C/N ratio. For the only significant SNP marker, the pairwise relationship between MAF and leaf nitrogen content in 2020, 2021 and 2022 was not significant (*p*‐value = 0.84; *p*‐value = 0.28; *p*‐value = 0.33). For the significant marker, the protein sequence of the protein PIR isoform X3 annotated in the genome assembly of *Q. lobata* Née (NCBI 2025) was annotated (NCBI 2025; UNiProt 2025). We recommend being cautious with these results; this one single observation, without any clear indication of a connection with leaf nitrogen content, could easily be a false positive observation. We would have expected at least a few significant overlapping markers between leaf nitrogen content and the C/N ratio, since the C/N ratio is calculated from leaf nitrogen content. Phenotypic variability of these traits may be too high to detect SNP‐trait associations across years (De La Torre et al. 2022), which can also be observed in Figure S1. Additionally, leaf nitrogen (N) content and the C/N ratio are influenced by complex physiological mechanisms (Gessler et al. 2017). These complex physiological mechanisms depend on the C and N availability before the drought event. Lower nutrient availability at the beginning of drought stress leads to a higher C/N ratio during drought, while higher nutrient availability leads to a lower C/N ratio during drought. Short and intense drought stress usually causes higher nitrogen depletion, while prolonged drought stress causes C starvation (Gessler et al. 2017). These complex physiological mechanisms may make association studies for these traits even more complicated. Additionally, stress levels may differ between years, and resultingly SNP‐trait associations are in fact environment‐dependent (see, e.g., Hrivnák et al. 2022).

Three SNP markers were associated with δ^13^C measured in 2020 at the permutation derived significance levels of *p* ≤ 0.000001. Only two SNP markers were identified as being associated with δ^13^C measured in 2021 and 2022. In the GWAS for δ^13^C measured in 2020 and 2022, we observed a peak on chromosome 10 that is overlapping with the peak observed in the GWAS for stomatal density on chromosome 10 from ~5.48 to ~13.67 Mb (Figures 1 and 2). This observation is meaningful as δ^13^C captures changes in carbon isotope composition due to stomatal closure as a result of reduced soil water availability and plant water status (Fotelli et al. 2003).

In general, all GWAS results should be considered carefully. SNP associations can be strongly influenced by the environment, since we worked with natural populations. And even though our sample size is already larger than in most GWAS in forest trees, and in a good range to detect marker‐trait associations (Hall et al. 2016), the validation of the markers in independent populations is still necessary before they can be used for stand selection and genetic population improvement. Furthermore, different soil properties were not considered, which may also have influenced the levels of drought stress. For future studies, we would recommend considering the role of different soil properties in the analysis or selecting populations growing on similar soils.

### 4.4 Results of the Polygenic Test $\hat{G}$ for δ ^13^C Reveal Signals of Polygenic Selection

Only a few or no significant SNP associations were observed in the GWAS for δ^13^C (Figure 2), leaf nitrogen and C/N ratio (Figure 5). Previous studies showed that it is difficult to identify significant markers for highly polygenic traits with infinitesimal sites contributing to the genetic variation (Beissinger et al. 2018; Zeng et al. 2018; Mahmoud et al. 2023). Previous studies assumed leaf nitrogen and carbon traits may have a polygenic basis (De La Torre et al. 2022). Therefore, we additionally applied the polygenic test $\hat{G}$ to our data set for the traits δ^13^C, leaf nitrogen and C/N ratio. According to $\hat{G}$, δ^13^C in 2020 and 2021, was under highly significant polygenic selection (*p* ~ 0) (see Table S24). In these years, we also observed the highest predictive ability of 0.8887 and 0.8840 on average based on our cross‐validations (see Figure S23). For the C/N ratio, no signals of polygenic selection were observed (Table S24). Negative selection with a pairwise relationship coefficient between allele effect estimates and allele frequency change of −0.0383 (*p* ~ 0.0014) was observed for leaf nitrogen measured in 2021, see Table S24. This year, the calculated predictive ability based on the cross‐validation varied the most among the 3 years. The lowest predictive ability observed in a cross‐validation was 0.77, and the highest was 0.92 (Figure S23). The detection of SNP markers and polygenic selection signals was not achieved for leaf nitrogen and carbon traits. The question remains whether the absence of polygenic selection signals is due to the previously mentioned complex physiological mechanisms of these traits. For future genomic prediction or $\hat{G}$ studies, we would recommend the usage of larger data sets in terms of individuals, because of the varying and partially low predictive abilities in our cross validations based on this data set.

In an environmental association analysis (EAA) conducted on the same genotyping data set as in this study, we did not observe any significant SNP associations on chromosome 10 (Tost et al. 2025). In the EAA, 273 markers on chromosome 2, spanning ~4.56 to ~16.27 Mb, appeared as a single broad peak (Tost et al. 2025). A more detailed comparison of the two studies can be found in Tost et al. (2025). Overall, there was no overlap between the SNP candidate markers from the two studies (Tost et al. 2025).

## 5 Conclusions

Our research provides insights into the genetic architecture of various drought‐stress traits in the studied population. We identified several very interesting SNPs for stomatal density, which should be evaluated in subsequent studies. Most of these candidate SNPs are located in one wide region on chromosome 10 from ~5.48 to ~13.67 Mb. Within this region, we found, based on a literature review, annotations for five genes with a direct impact on stomatal density, which comprise *aluminum‐activated malate transporter 2‐like*, *transcription factor MYB35‐like, ATP‐dependent Clp protease adapter protein ClpS1, Aspartic protease in guard cell 1* and *BONZAI1*. The minor allele frequencies observed at the candidate SNP markers in this region are positively related to the mean stomatal density per stand, altitude and precipitation and negatively related to temperature. Therefore, we can deduce that this entire region is important for local adaptation. Furthermore, we identified one candidate SNP associated with leaf nitrogen content. For δ^13^C measured in 1 year, we identified two significant SNP marker associations, also located within the wide peak region on chromosome 10 from ~5.48 to ~13.67 Mb associated with stomatal density. We also observed signals of highly polygenic selection for δ^13^C. In conclusion, our research demonstrates that polygenic tests like $\hat{G}$ and GWAS can complement each other. $\hat{G}$ focuses on the small effect loci and uncovers their combined effect on a trait. Our GWAS performed very well in identifying a candidate region for further studies to identify SNP candidates for stomatal density. However, it did return only a limited number of SNP markers associated with δ^13^C in 2020, but the polygenic test $\hat{G}$ returned signals of polygenic selection for this trait. Our results suggest that traits such as δ^13^C are polygenic, whereas stomatal density may be influenced by a few large‐effect loci. Therefore, genomic prediction seems to be a promising tool for putatively highly polygenic traits like δ^13^C. Due to limited knowledge about the genetic basis of many traits studied in forest species, it is recommended for future research to combine genome‐wide association studies, polygenic tests and genomic prediction.

## Author Contributions

Mila Tost wrote the initial draft, reviewed, and edited the manuscript under supervision of Oliver Gailing with help from Henning Wildhagen, Timothy Beissinger, Markus Müller, and Alexandru Lucian Curtu. Ourania Grigoriadou‐Zormpa, Mehdi Ben Targem, and Alexandru Lucian Curtu conducted and organized the leaf sample and data collection. The analysis was conducted by Mila Tost and Selina Wilhelmi The visualization of the data was done by Mila Tost and Ourania Grigoriadou‐Zormpa. Oliver Gailing, Alexandru Lucian Curtu, and Henning Wildhagen planned and conceptualized the study. Oliver Gailing and Henning Wildhagen acquired financial support for the project and oversaw the project administration. Oliver Gailing, Alexandru Lucian Curtu, Henning Wildhagen, Timothy Beissinger, and Markus Müller developed the design and methodology used in this study.

## Funding

This work was financially supported by the Federal Ministry of Food and Agriculture—FNR‐Waldklimafonds (Reference numbers: 2218WK43B4, 2218WK43A4). The drought markers project is a collaboration between the University of Goettingen (Germany), the HAWK (Germany), the NW‐FVA (Germany), and Transilvania University of Brasov (Romania). We utilized the computational resources of the University of Goettingen's GWDG. Open Access funding enabled and organized by Projekt DEAL.

## Supporting information


**File S1:** Phenotypic trait measurements: diameter at breast height (DBH), stomatal density, leaf carbon content measured in 2020, 2021, 2022, leaf nitrogen content measured in 2020, 2021 2022, C/N ratio calculated for 2020, 2021, 2022, and water use efficiency as δ13C measured in 2020, 2021, 2022.
**S2.** Correlations of all phenotypic traits and their correlation coefficients (*p* ≤ 0.05).
**S3.** Correlations of the traits measured in different years for leaf nitrogen content measured in 2020, 2021 2022, C/N ratio calculated for 2020, 2021, 2022, and water use efficiency as δ13C measured in 2020, 2021, 2022.
**Table S4:** Overview over trait examined, years of sampling, number of sampled trees, number of significant markers detected, chromosomal locations of markers.
**S5.** Results from the principal component analysis (PCA) based on the neutral markers with SNPrelate (Zheng et al. 2012) with (a) the eigenvalues of principal components (PC), (b) the first PC plotted against the second PC, (c, d). The first two PCs explained ~12.81% was used in the genome‐wide association study (GWAS) as covariate.
**S6.** Average monthly temperature in °C and monthly precipitation (mm) measured during the sampling season from April to September in 2020, 2021, and 2022 in Ruia (a), Lupului (b), Solomon (c), Tampa (d), and Lempes (e), which were used in the environmental principal component analysis (PCA).
**S7.** Average monthly temperature in °C and monthly precipitation (mm) measured during the sampling season from April to September in 2020, 2021, and 2022 in Ruia, Lupului, Solomon, Tampa, and Lempes, which were used in the environmental principal component analysis (PCA).
**S8.** Results from the principal coordinate analysis (PCoA) on precipitation and temperature, of the sampling years 2020 (a) and the eigenvalues of PCs (b), 2021 (c and d) and 2022 (e and f). Environmental PC 1 and Environmental PC 2, which explained ~100% were used in the genome‐wide association study (GWAS) as covariate.
**S9.** Genomic relationship matrix calculated with GCTA (Yang et al. 2011) which shows the pairwise relationship coefficients between all individuals and the inbreeding coefficient of the individuals on the main diagonal.
**S10.** Distribution of pairwise relationship coefficients between all individuals. The peak around 0 indicates no relationship. The dashed lines show the relationship coefficient (relatedness) of 0.1 and 0.25. The line at the relationship coefficient (relatedness) of 0.5 shows the cutoff threshold for individuals used in the GWAS.
**S11.** QQ‐Plots of all phenotypic variables. The QQ plots are following this order: Stomatal density (a), δ13C measured in 2020 (b), δ13C measured in 2021 (c), δ13C measured in 2022 (d), leaf nitrogen (e) and C/N ratio (f).
**S12.** List of remaining candidate SNPs associated with stomatal density (SD) and δ13C measured in 2020 with premutation derived significance levels of *p* ≤ 0.000001 and their annotated gene variants, the underlying genes with their locations, functions and predicted impact (LOW or MODIFIER (not predicted)) based on SnpEff (Cingolani et al. 2012).
**S13.** Coding region of the gene variant Bhaga_10.g917 (ALMT2) and the significant marker on chromosome 10 at ~7.56 Mb in blue and pairwise LD heat map from 7.550433 to 7,560,433 Mb.
**S14.** Coding region of the gene variant Bhaga_10.g957 (MYB35‐like) and the five significant markers on chromosome 10 at ~7.78 to 7.82 Mb (7,785,927; 7,785,978; 7,787,468; 7,820,271; 7,820,389) Mb in blue and pairwise LD heat map from 7.765927 to 7.840389 Mb.
**S15.** Coding region of the gene variants Bhaga_10.g1460 (ClpS1), Bhaga_10.g1462 (ASPG1), and the significant marker on chromosome 10 at ~11.9 Mb in blue and pairwise LD heat map from 11.888957 to 11.918957 Mb.
**S17.** List of other genes close to markers associated with stomatal density but without a direct impact on stomatal density based on the literature review
**S18.** Minor allele frequency (MAF) observed in the different stands at the significant markers (*p* ≤ 0.000001) on chromosome 10 associated with stomatal density and the number of heterozygous individuals (Nr of Het), number of homozygous individuals (Nr of Hom) and the total number of genotyped individuals (Nr of total ind.).
**S19.** Correlation between stomatal density and the minor allele frequency (MAF) of the significant markers on chromosome 1
**S20.** Minor allele frequency (MAF) observed in the different stands at the significant markers (*p* ≤ 0.000001) associated with leaf nitrogen content, the stand mean, the number of heterozygous individuals (Nr of Het) and the total number of genotyped individuals (Nr of total ind.).
**S21.** Correlation between leaf nitrogen content measured in 2021 and the minor allele frequency (MAF) of the significant marker (*p* ≤ 0.000001) on chromosome 2 at ~31.804 Mb. This marker was annotated with the gene variant Bhaga_2.g3457 with the underlying gene XP_030960280.1, also known as abscisic‐aldehyde oxidase‐like.
**S22.** Rate of false positive observations observed in Ghat test calculated based on the 100 randomly generated traits with 10 replications (10 replications × 100 random traits).
**S23.** Results from five‐fold cross‐validation with the mean predictive ability as 𝑟𝑦𝑔̂ (correlation of predicted trait estimates and observed trait measurements) with random partition of stands in test and training set (a) and fivefold cross‐validation in which one stand was left out and the remaining stands were used for prediction (b) with 100 replications based on RR‐BLUP for the real trait measurements.
**S24.** Results from 𝐺̂ test with change calculated between groups (Ruia, Lupului vs. Tampa, Lempes) calculated between groups with 100,000 permutations and 7000 effective markers calculated based on the previous test runs for carbon isotope composition δ13C.
**S25.** Observed codon changes which lead to an amino acid (AA) replacement and a resulting change in polar bonds (hydrophobic or hydrophilic) at the underlying gene variants of markers associated with stomatal density
**S26.** Genes and their functional categories of genes derived from the KEGG database (2025) and the level of significance shown as −log10(*p*) of the underlying SNP markers from the GWAS for stomatal density.
**S27.** Correlation between stomatal density and observed genotypes at the significant marker (*p* ≤ 0.000001) on chromosome 10 at ~7.82 Mb. The coefficient of determination *R* ^2^ was 0.2353 (*p* ~ 0). This marker is close to the coding region of the gene variant Bhaga_10.g957 (MYB35‐like)

## Data Availability

The used data comes from the “Droughtmarkers” project (Reference numbers: 2218WK43B4, 2218WK43A4). The data is publicly available at figshare: 10.6084/m9.figshare.28748924. Our analysis code is publicly available on github: https://github.com/MilaTost/DroughtMarkers.
