# Supplementary material for: Genome‐Wide Association Study in European Beech ( Fagus sylvatica L.) for Drought Stress Related Traits
Source: Physiol Plant. 2026 Sep 22;178(5):e71120. doi: 10.1111/ppl.71120 (PMC13598277; doi:10.1111/ppl.71120)

Supplementary files

Genome-wide association study in European beech (*Fagus sylvatica* L.) for drought stress related traits

Authors: Mila Tost, Ourania Grigoriadou-Zormpa, Selina Wilhelmi, Timothy Beissinger, Mehdi Ben Targem, Markus Müller, Henning Wildhagen, Alexandru Lucian Curtu, Oliver Gailing

S1: Phenotypic trait measurements: diameter at breast height (DBH), stomatal density, leaf carbon content measured in 2020, 2021, 2022, leaf nitrogen content measured in 2020, 2021 2022, C/N ratio calculated for 2020, 2021, 2022 and water use efficiency as $\delta^{13}C$ measured in 2020, 2021, 2022.

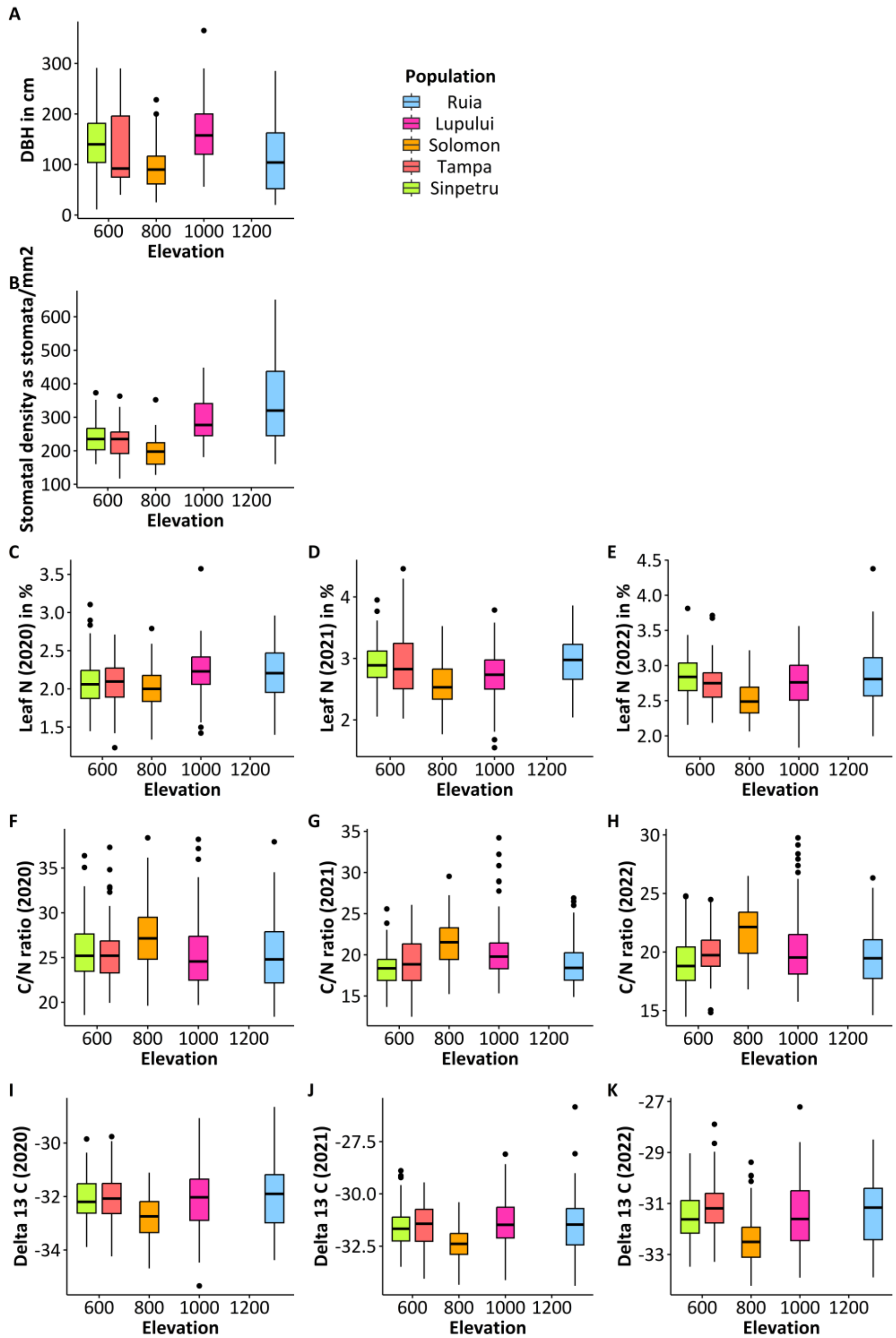

S2: Correlations of all phenotypic traits and their correlation coefficients ($p \leq 0.05$).

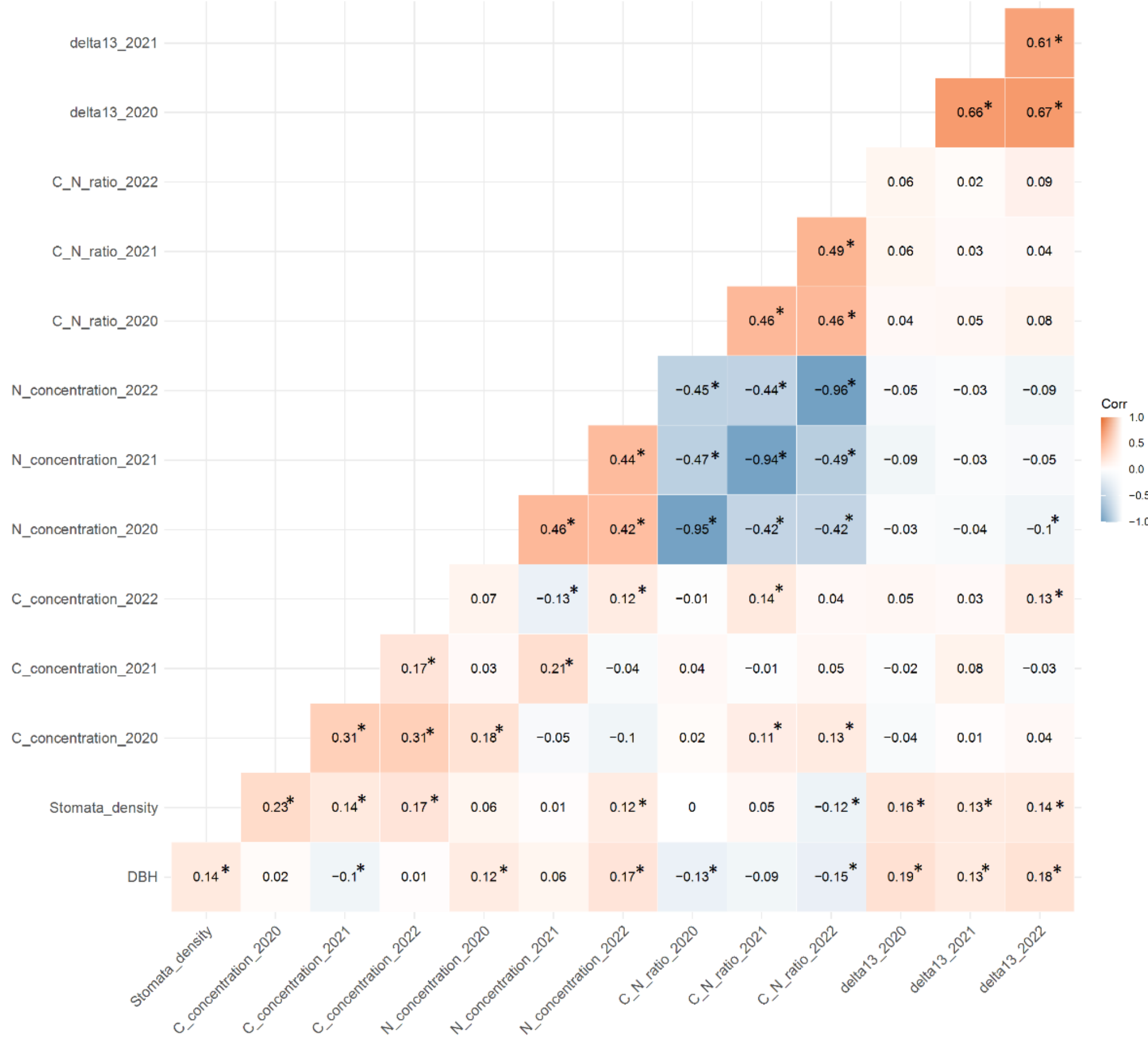

S3: Correlations of the traits measured in different years for leaf nitrogen content measured in 2020, 2021 2022, C/N ratio calculated for 2020, 2021, 2022 and water use efficiency as δ^{13}C measured in 2020, 2021, 2022.

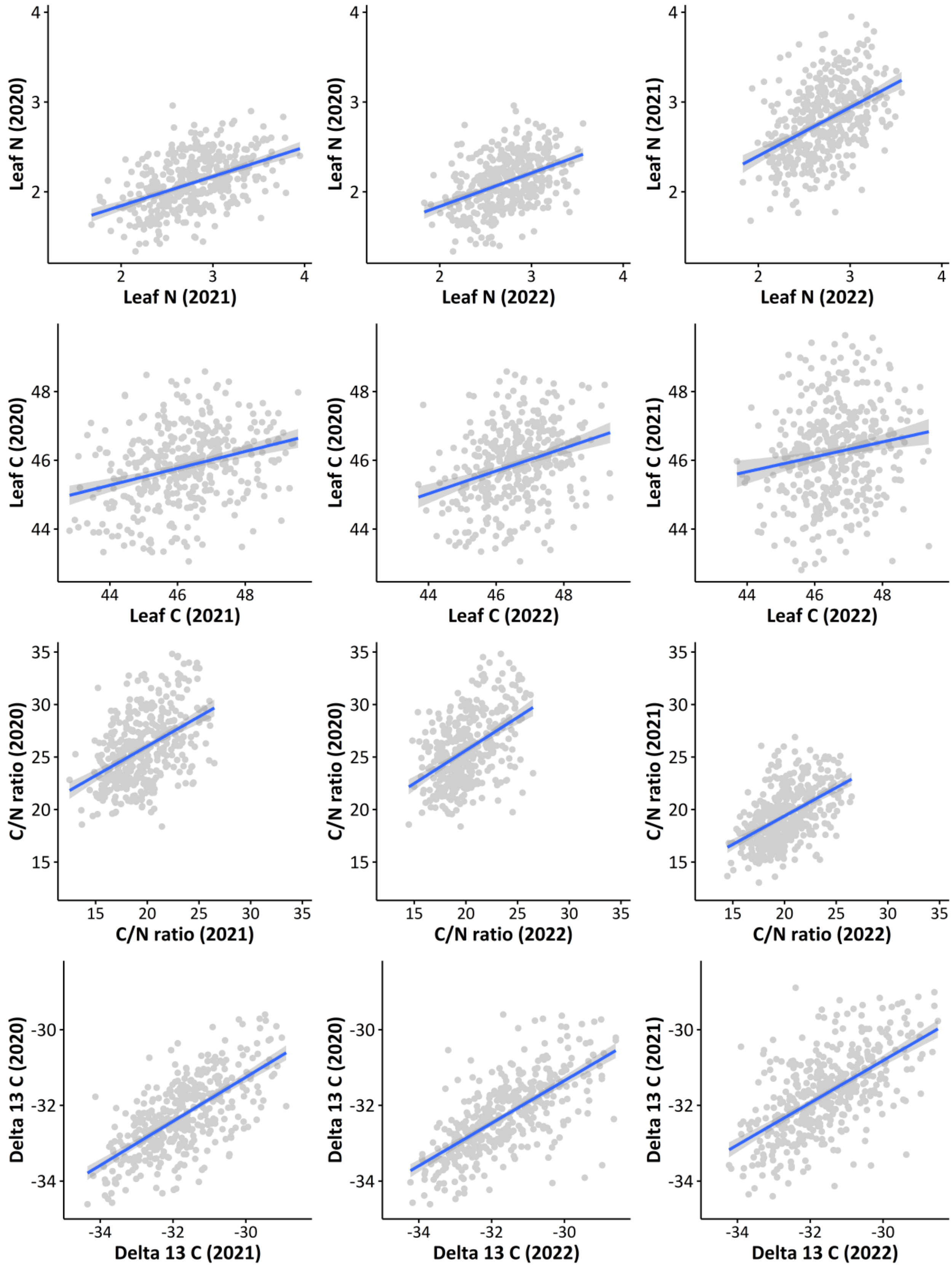

S4: Overview over trait examined, years of sampling, number of sampled trees, number of significant markers detected, chromosomal locations of markers.

	**Number of phenotyped samples**			**Number of markers used in GWAS**	**Chromosomal locations of markers**
**Year**	2020	2021	2022	610678	1, 2, 3, 4, 5, 6, 7, 8, 9, 10, 11, 12
**Trait**					
Stomatal density	NA	314	NA		
$\delta^{13}C$	444	457	454		
Leaf N	451	457	450		
C/N ratio	440	454	450		

S5: Results from the principal component analysis (PCA) based on the neutral markers with SNPrelate (Zheng et al., 2012) with **a**) the eigenvalues of principal components (PC), **b**) the first PC plotted against the second PC, **c**), **d**). The first two PCs explained ~12.81% was used in the genome-wide association study (GWAS) as covariate.

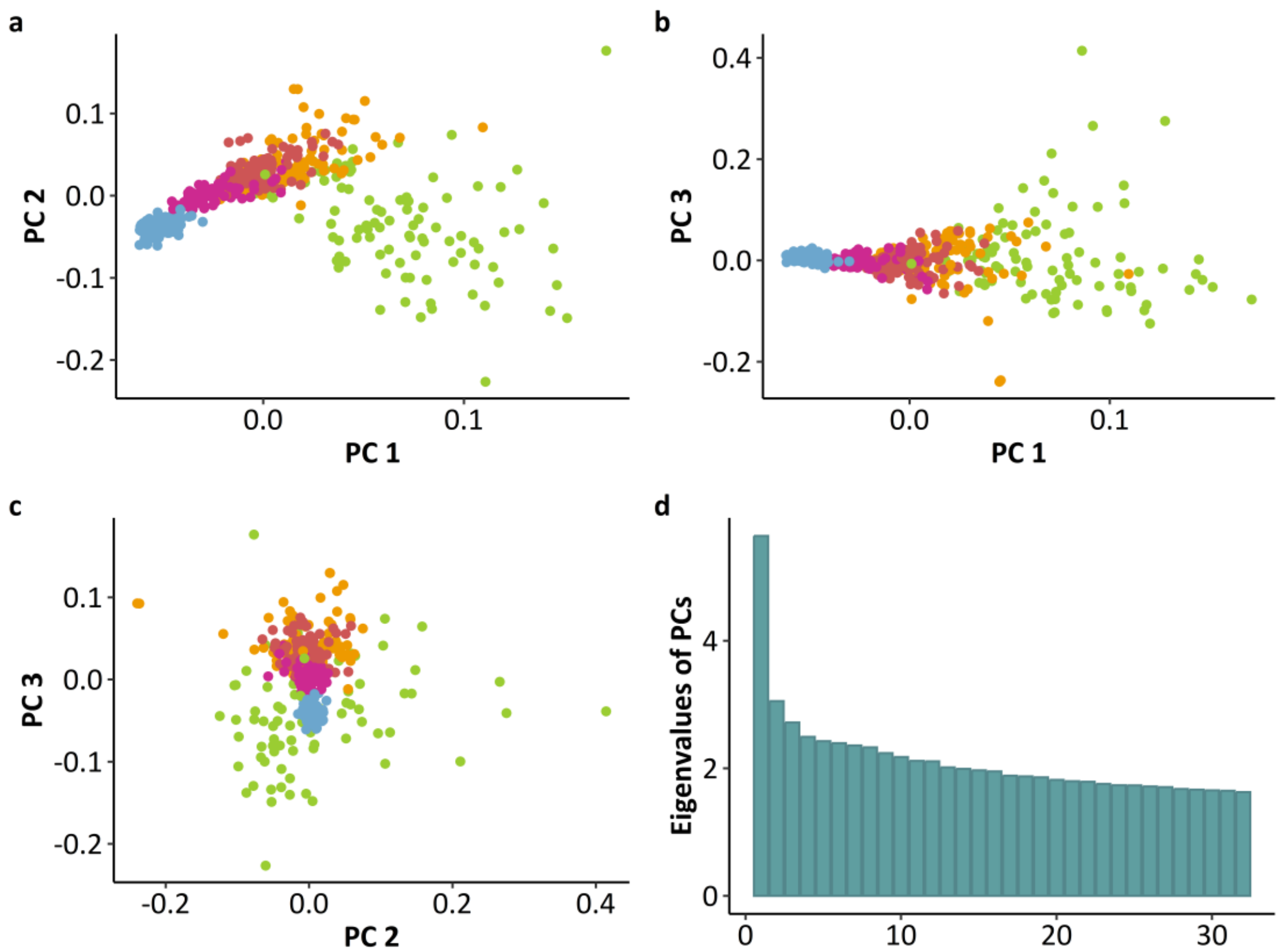

S6: Average monthly temperature in °C and monthly precipitation (mm) measured during the sampling season from April to September in 2020, 2021 and 2022 in Ruia (a), Lupului (b), Solomon (c), Tampa (d) and Lempes (e), which were used in the environmental principal component analysis (PCA).

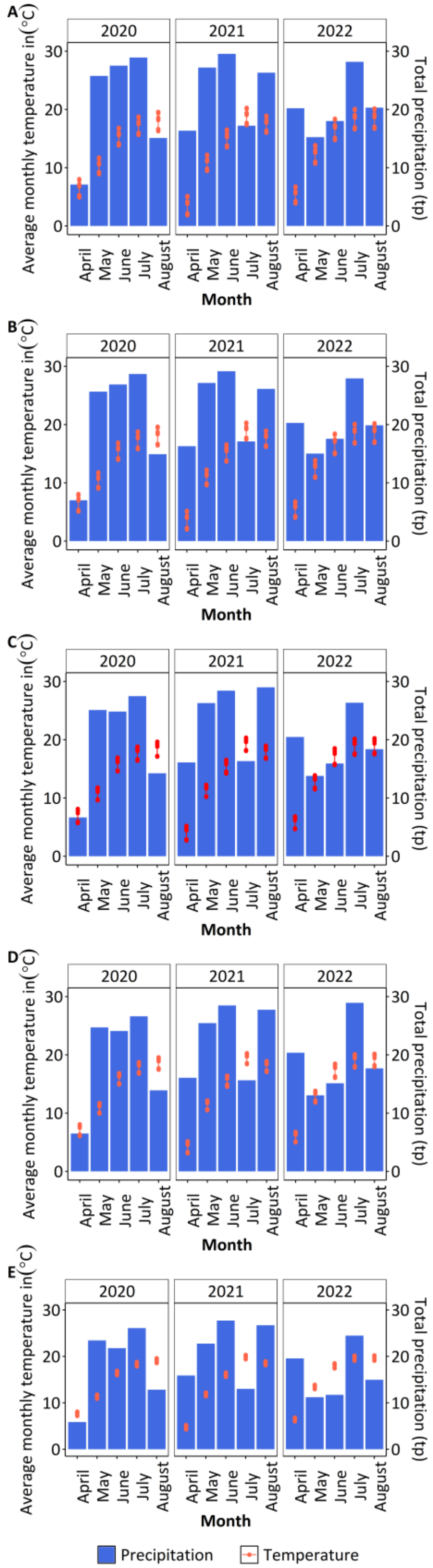

S7: Average monthly temperature in °C and monthly precipitation (mm) measured during the sampling season from April to September in 2020, 2021 and 2022 in Ruia, Lupului, Solomon, Tampa and Lempes, which were used in the environmental principal component analysis (PCA).

Stand	Year	Stand	Precipitation in mm	Temperature in °C
April	2020	Ruia	800.81	6.68
April	2020	Lupului	789.86	6.79
April	2020	Solomon	749.71	7.16
April	2020	Tampa	732.19	7.32
April	2020	Lempes	659.19	7.79
May	2020	Ruia	2861.424	10.49
May	2020	Lupului	2851.752	10.58
May	2020	Solomon	2789.256	10.92
May	2020	Tampa	2745.36	11.06
May	2020	Lempes	2604.744	11.48
June	2020	Ruia	3099.58	15.52
June	2020	Lupului	3028.04	15.62
June	2020	Solomon	2797.36	15.98
June	2020	Tampa	2715.6	16.14
June	2020	Lempes	2452.8	16.60
July	2020	Ruia	3718.512	17.40
July	2020	Lupului	3686.52	17.51
July	2020	Solomon	3539.208	17.88
July	2020	Tampa	3433.56	18.03
July	2020	Lempes	2899.368	18.48
August	2020	Ruia	1701.63	18.11
August	2020	Lupului	1681.19	18.23
August	2020	Solomon	1603.81	18.62
August	2020	Tampa	1566.58	18.77
August	2020	Lempes	1446.13	19.23
April	2021	Ruia	1877.112	3.70
April	2021	Lupului	1869.672	3.82
April	2021	Solomon	1848.84	4.22
April	2021	Tampa	1843.632	4.39
April	2021	Lempes	1823.544	4.91
May	2021	Ruia	2964.53	11.02
May	2021	Lupului	2958.69	11.11
May	2021	Solomon	2865.98	11.44
May	2021	Tampa	2773.27	11.57
May	2021	Lempes	2481.27	11.95
June	2021	Ruia	3392.64	15.22
June	2021	Lupului	3348	15.33
June	2021	Solomon	3263.184	15.67
June	2021	Tampa	3270.624	15.79
June	2021	Lempes	3181.344	16.18
July	2021	Ruia	1876.83	18.99
July	2021	Lupului	1865.88	19.09
July	2021	Solomon	1780.47	19.44
July	2021	Tampa	1703.09	19.59
July	2021	Lempes	1418.39	20.05
August	2021	Ruia	4059.264	17.67
August	2021	Lupului	4029.504	17.77
August	2021	Solomon	3828.624	18.10
August	2021	Tampa	3667.176	18.24
August	2021	Lempes	3068.256	18.68
April	2022	Ruia	2276.14	5.52
April	2022	Lupului	2285.63	5.61
April	2022	Solomon	2305.34	5.95
April	2022	Tampa	2295.12	6.10
April	2022	Lempes	2203.87	6.53

May	2022	Ruia	1694.832	12.50
May	2022	Lupului	1668.048	12.61
May	2022	Solomon	1531.152	12.98
May	2022	Tampa	1449.312	13.12
May	2022	Lempes	1243.968	13.57
June	2022	Ruia	2027.94	16.78
June	2022	Lupului	1977.57	16.91
June	2022	Solomon	1792.88	17.34
June	2022	Tampa	1702.36	17.53
June	2022	Lempes	1321.3	18.12
July	2022	Ruia	3592.776	18.52
July	2022	Lupului	3560.784	18.64
July	2022	Solomon	3361.392	19.06
July	2022	Tampa	3215.568	19.24
July	2022	Lempes	2720.808	19.78
August	2022	Ruia	2287.09	18.63
August	2022	Lupului	2238.18	18.76
August	2022	Solomon	2070.28	19.16
August	2022	Tampa	1992.17	19.33
August	2022	Lempes	1684.84	19.85

S8: Results from the principal coordinate analysis (PCoA) on precipitation and temperature, of the sampling years 2020 (**a**) and the eigenvalues of PCs (**b**), 2021 (**c** and **d**) and 2022 (**e** and **f**). Environmental PC 1 and Environmental PC 2, which explained ~100% were used in the genome-wide association study (GWAS) as covariate.

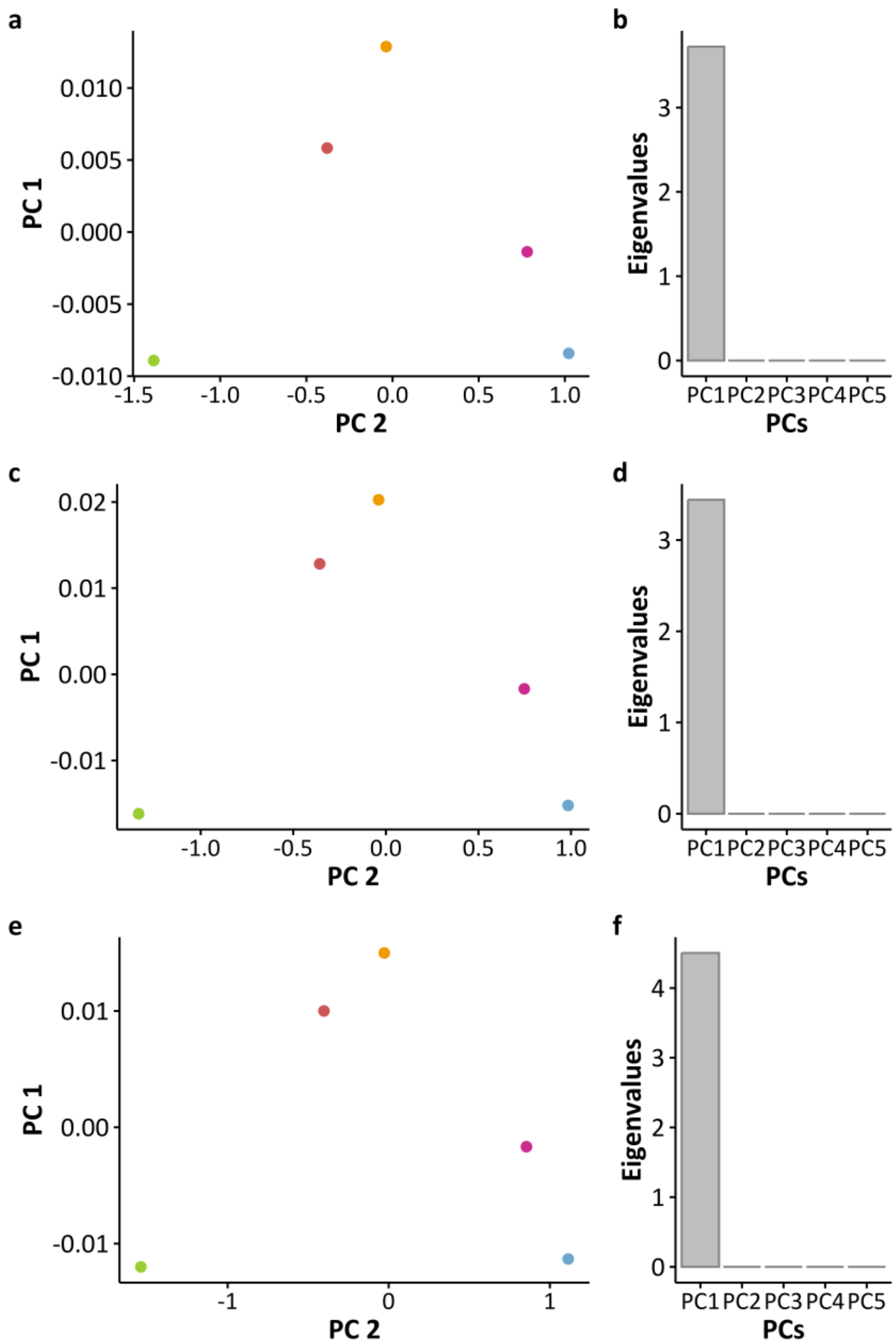

S9: Genomic relationship matrix calculated with GCTA (Yang et al. 2011) which shows the pairwise relationship coefficients between all individuals and the inbreeding coefficient of the individuals on the main diagonal.

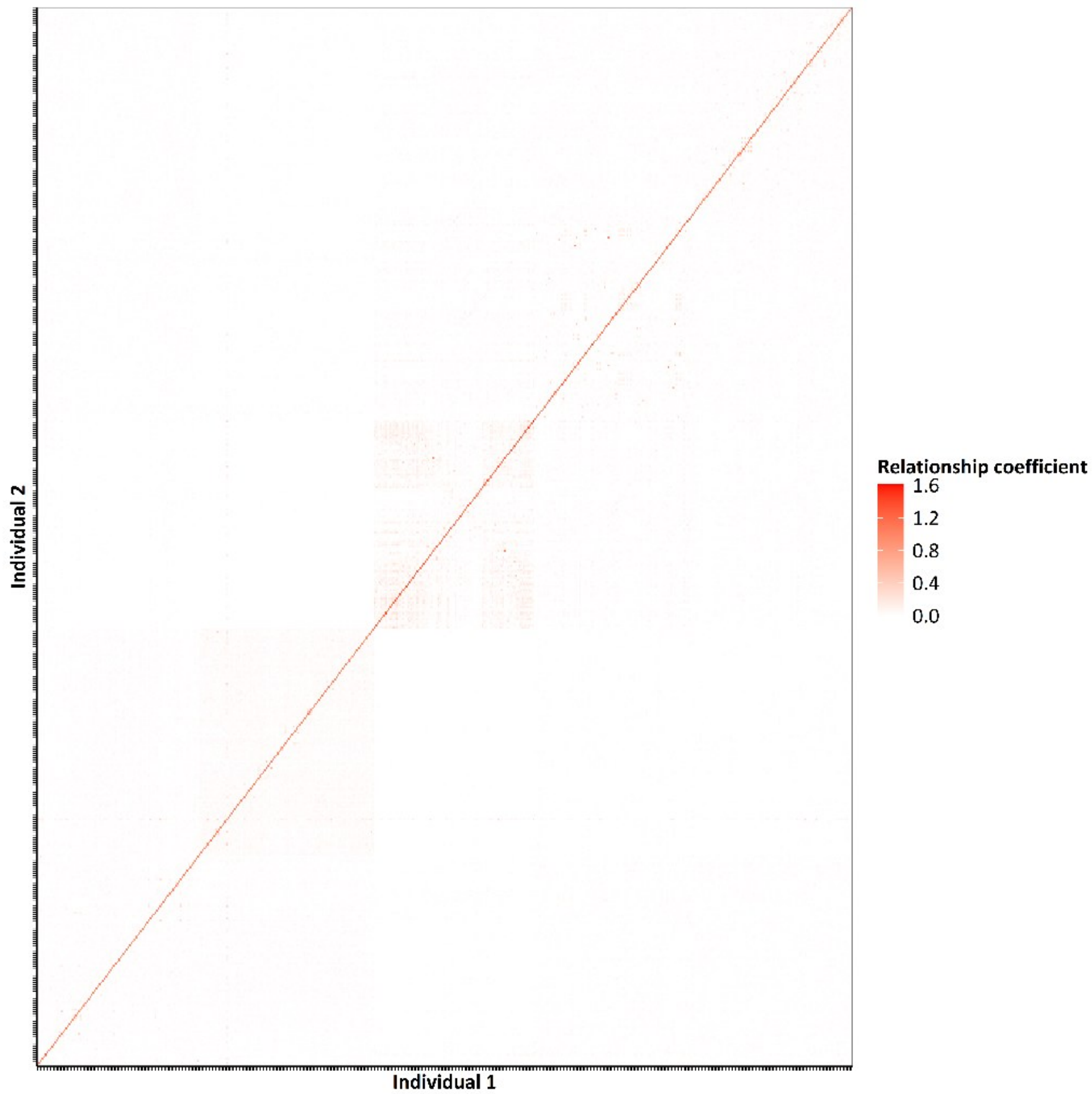

S10: Distribution of pairwise relationship coefficients between all individuals. The peak around 0 indicates no relationship. The dashed lines show the relationship coefficient (relatedness) of 0.1 and 0.25. The line at the relationship coefficient (relatedness) of 0.5 shows the cutoff threshold for individuals used in the GWAS.

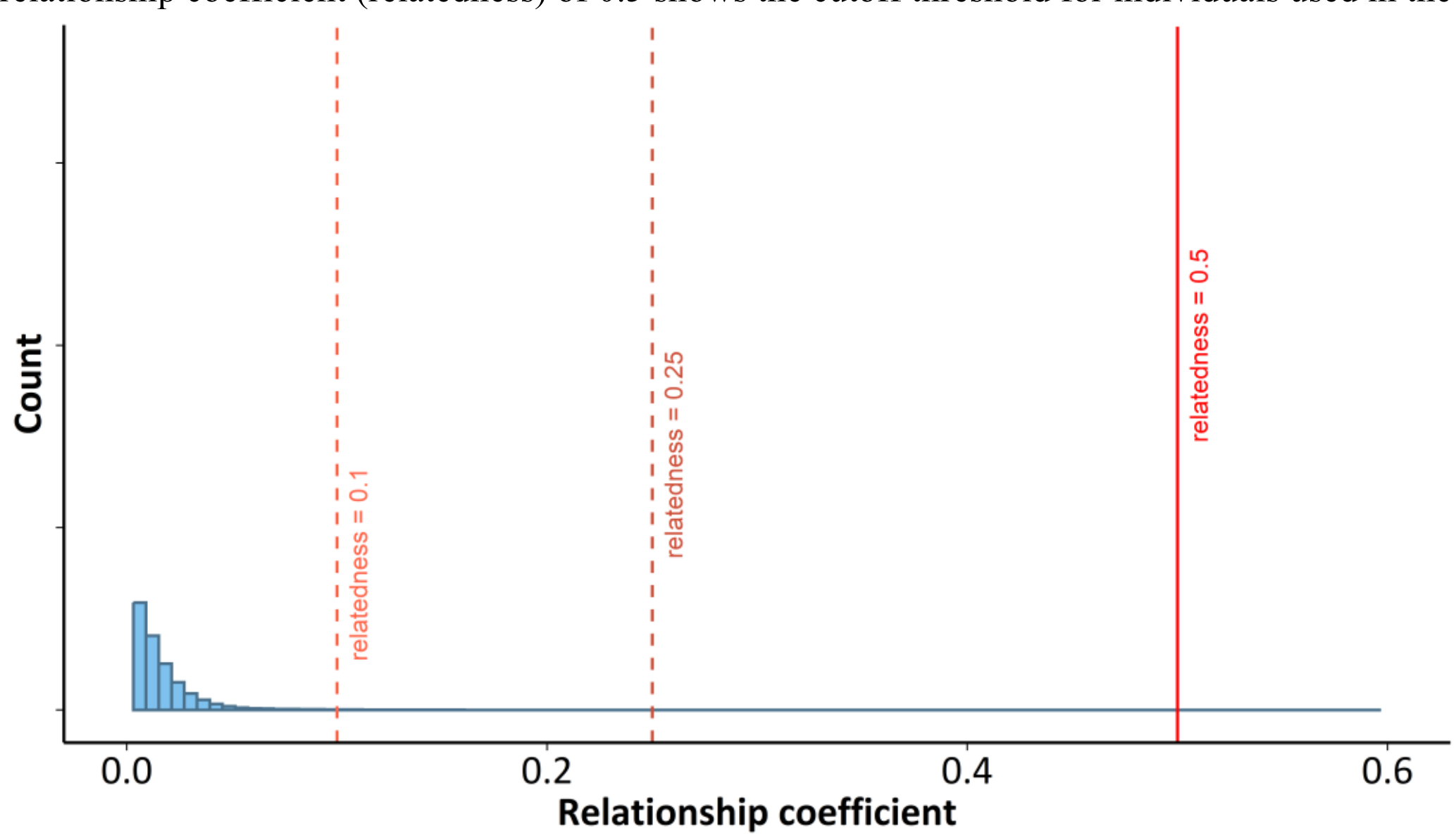

S11: QQ-Plots of all phenotypic variables. The QQ plots are following this order: Stomatal density (**a**), δ^{13}C measured in 2020 (**b**), δ^{13}C measured in 2021 (**c**), δ^{13}C measured in 2022 (**d**), leaf nitrogen (**e**) and C/N ratio (**f**).

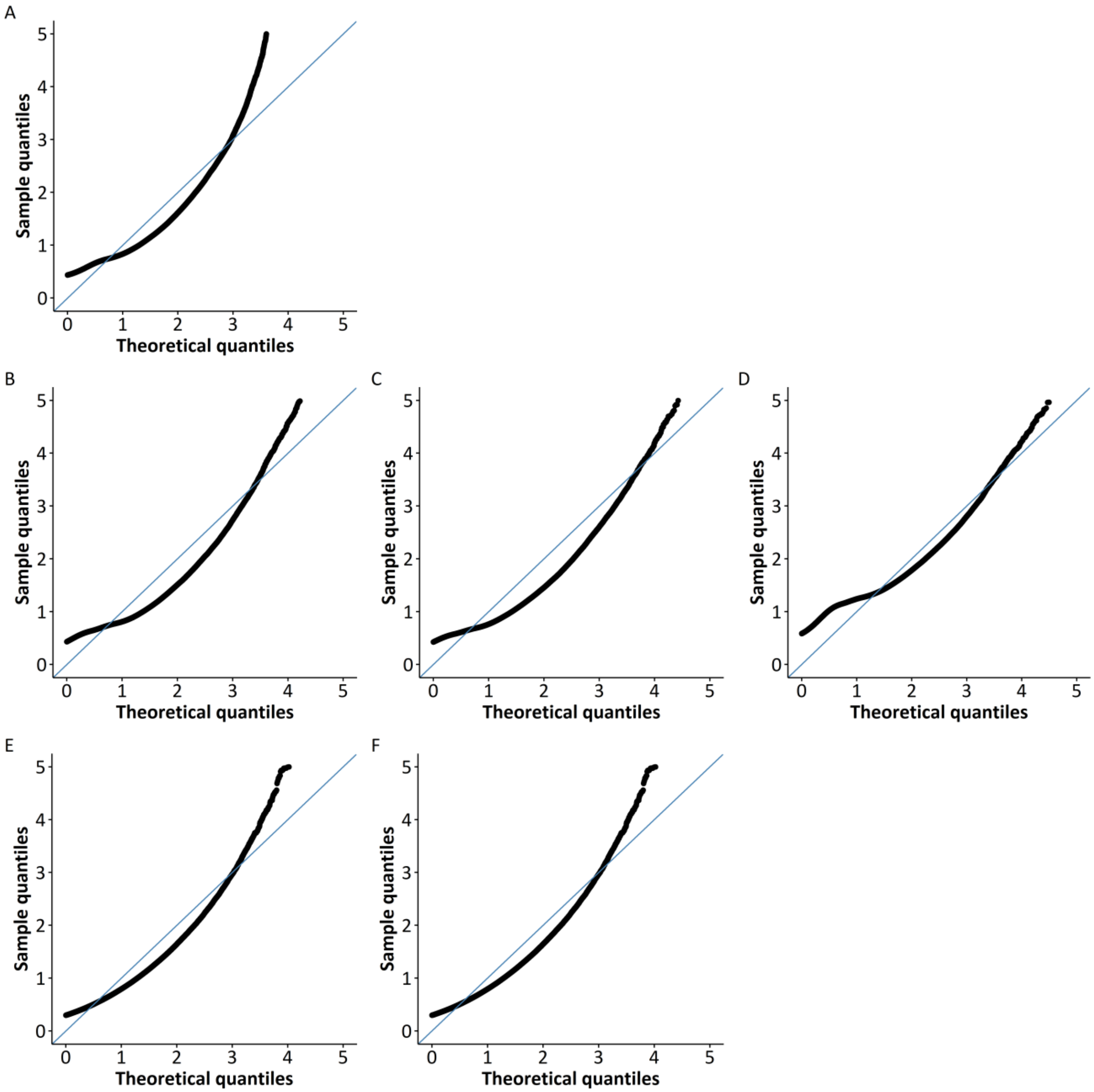

S12: List of remaining candidate SNPs associated with stomatal density (SD) and $\delta^{13}C$ measured in 2020 with premutation derived significance levels of p ≤0.000001 and their annotated gene variants, the underlying genes with their locations, functions and predicted impact (LOW or MODIFIER (not predicted)) based on SnpEff (Cingolani et al. 2012).

Trait	Chr	Position of SNP	Distance to gene variant in kb	Gene variant	Gene
SD	4	40808183	NA	NA	NA
		40808384	NA	NA	NA
		40816400	NA	NA	NA
		40818194	NA	NA	NA
		40854731	NA	NA	NA
	5	40871447	NA	NA	NA
		40871445	NA	NA	NA
		40871457	NA	NA	NA
	6	15825445	NA	NA	NA
		15825457	NA	NA	NA
	8	7588414	1.9	Bhaga_8.g971	NA
		7588499	2.522	Bhaga_8.g971	NA
			2.505	Bhaga_8.g971	NA
			2.206	Bhaga_8.g970	NA
		17371189	NA	NA	NA
	9	14893568	~-6.168 to -6.31	Bhaga_9.g1841	TYK12172.1
			~6.161	Bhaga_9.g1841	TYK12172.1
			~6.207	Bhaga_9.g1841	TYK12172.1
	10	5475876	1.458	Bhaga_10.g666	NA
		5889667	-2.283	Bhaga_10.g707	NA
		5889683	-2.299	Bhaga_10.g707	NA
		5889667	-2.239	Bhaga_10.g707	NA
		5889683	-2.255	Bhaga_10.g707	NA
		6025543	NA	NA	NA
		6851324	0.609	Bhaga_10.g818	NA
		7513203	-5.776	Bhaga_10.g911	NA
		7513203	-5.743	Bhaga_10.g911	NA
		7513203	2.289	Bhaga_10.g913	NA
		7513203	2.327	Bhaga_10.g913	NA
		7598413	5.904	Bhaga_10.g925	NA
		7696317	-5.333	Bhaga_10.g940	NA
		7825443	-9.178	Bhaga_10.g958	NA
			3.498	Bhaga_10.g960	NA
		8223372	-4.314	Bhaga_10.g1011	NA
		8223513	-4.455	Bhaga_10.g1011	NA
		8223537	-4.479	Bhaga_10.g1011	NA
		8223633	-4.575	Bhaga_10.g1011	NA
		8260462	-1.878	Bhaga_10.g1017	NA
		8260475	-1.891	Bhaga_10.g1017	NA
		8260485	-1.901	Bhaga_10.g1017	NA
		8260499	-1.915	Bhaga_10.g1017	NA
		8323901	-9.911	Bhaga_10.g1028	NA
		8336012	-9.97	Bhaga_10.g1030	NA
		8323901	2.167	Bhaga_10.g1030	NA
		8323901	3.903	Bhaga_10.g1031	NA
		8336012	-8.208	Bhaga_10.g1031	NA
		8336012	-0.523	Bhaga_10.g1033	NA
		8336012	-0.494	Bhaga_10.g1033	NA
		8336012	-0.443	Bhaga_10.g1033	NA

		8336012	1.204	Bhaga_10.g1033	NA
		8336012	1.207	Bhaga_10.g1033	NA
		8399367	7.189	Bhaga_10.g1041	NA
		8399422	7.134	Bhaga_10.g1041	NA
	10	8399501	9.833	Bhaga_10.g1040	NA
		8524101	-3.278	Bhaga_10.g1057	NA
			7.348	Bhaga_10.g1059	NA
		8895976	-4.667	Bhaga_10.g1101	NA
		8895999	-4.69	Bhaga_10.g1101	NA
		9512768	-5.09	Bhaga_10.g1186	NA
		8399501	9.833	Bhaga_10.g1040	NA
		8524101	-3.278	Bhaga_10.g1057	NA
		8895976	-4.667	Bhaga_10.g1101	NA
		8895999	-4.69	Bhaga_10.g1101	NA
		9512768	-5.09	Bhaga_10.g1186	NA
		10058669	-6.538	Bhaga_10.g1261	NA
		10058669	7.698	Bhaga_10.g1263	NA
		10784123	-4.062	Bhaga_10.g1335	NA
		10805547	-4.365	Bhaga_10.g1336	NA
		10805606	-4.424	Bhaga_10.g1336	NA
		10806739	-5.557	Bhaga_10.g1336	NA
		10806877	-5.695	Bhaga_10.g1336	NA
$\delta^{13}C$ (2020)	10	10907107	-3.796	Bhaga_10.g1348	XP_030940353.1
		10907267	-3.643	Bhaga_10.g1348	
SD	10	10909086	-1.853	Bhaga_10.g1353	
		10909126	~-18.6 to -18.4	Bhaga_10.g1353	PQQ02746.1
		10909168			
		10909306			
		10909343			
		10915528	2.844	Bhaga_10.g1350	XP_030941044.1
		11233527	~-9.6 to -11.4	Bhaga_10.g1390	XP_023922841.1
		11233527			
		11233581			
		11401610	NA	NA	NA
		11467008	NA	NA	NA
		11467225	NA	NA	NA
		11511420	~6.9 to 10.4	Bhaga_10.g1422	XP_023884902.1
			~-11.4	Bhaga_10.g1424	KAB1212694.1
		11599598	NA	NA	NA
		11634927	NA	NA	NA
		11988800	NA	NA	NA
		12053423	NA	NA	NA
		12684288	NA	NA	NA
$\delta^{13}C$ (2020)	11	33542927	NA	NA	NA

S13: Coding region of the gene variant *Bhaga_10.g917* (*ALMT2*) and the significant marker on chromosome 10 at ~7.56 Mb in blue and pairwise LD heat map from 7.550433 to 7560433 Mb.

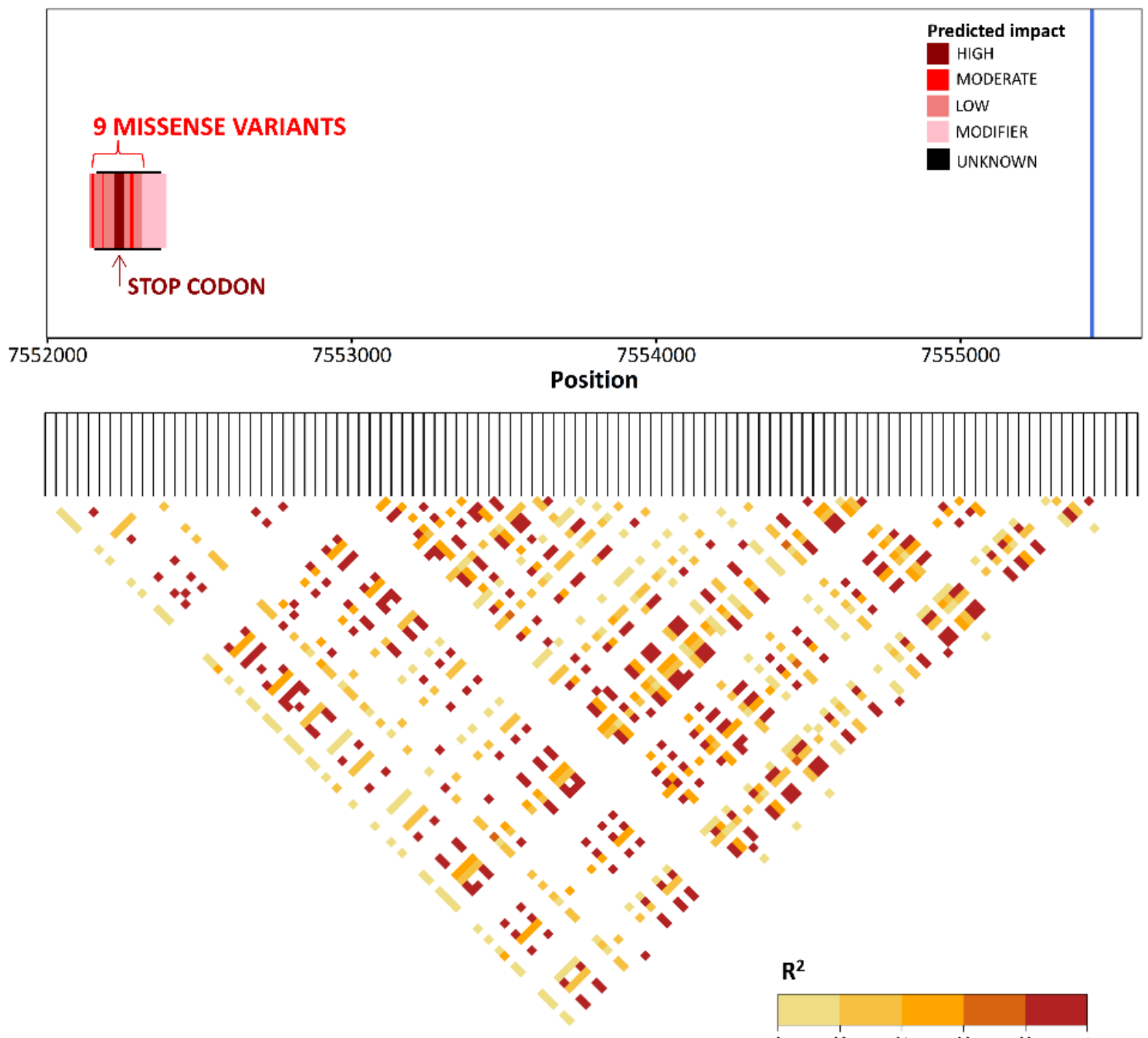

S14: Coding region of the gene variant *Bhaga_10.g957* (*MYB35-like*) and the five significant markers on chromosome 10 at ~7.78 to 7.82 Mb (7785927; 7785978; 7787468; 7820271; 7820389) Mb in blue and pairwise LD heat map from 7.765927 to 7.840389 Mb.

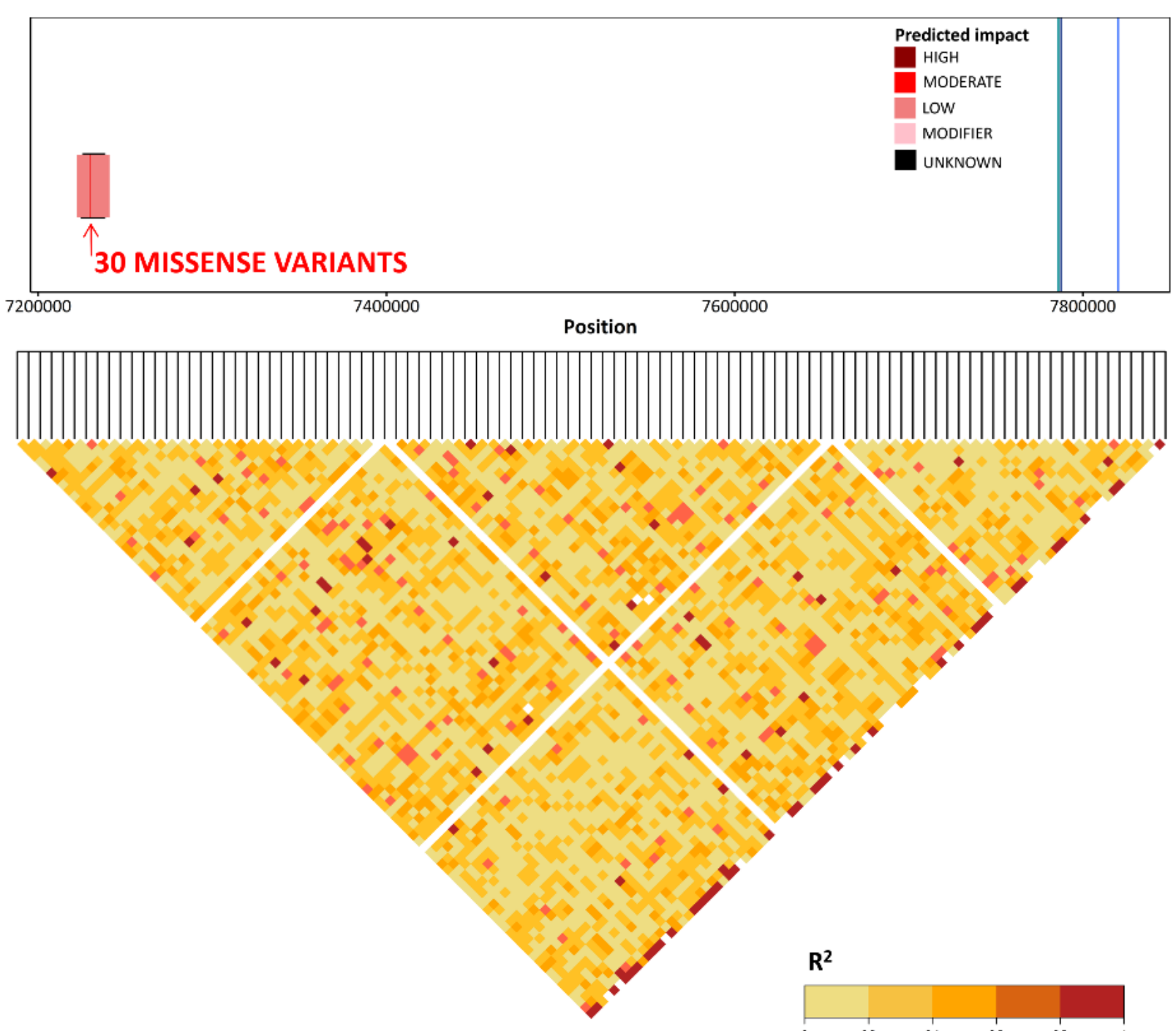

S15: Coding region of the gene variants *Bhaga_10.g1460 (ClpS1)*, *Bhaga_10.g1462* (*ASPG1*) and the significant marker on chromosome 10 at ~11.9 Mb in blue and pairwise LD heat map from 11.888957 to 11.918957 Mb.

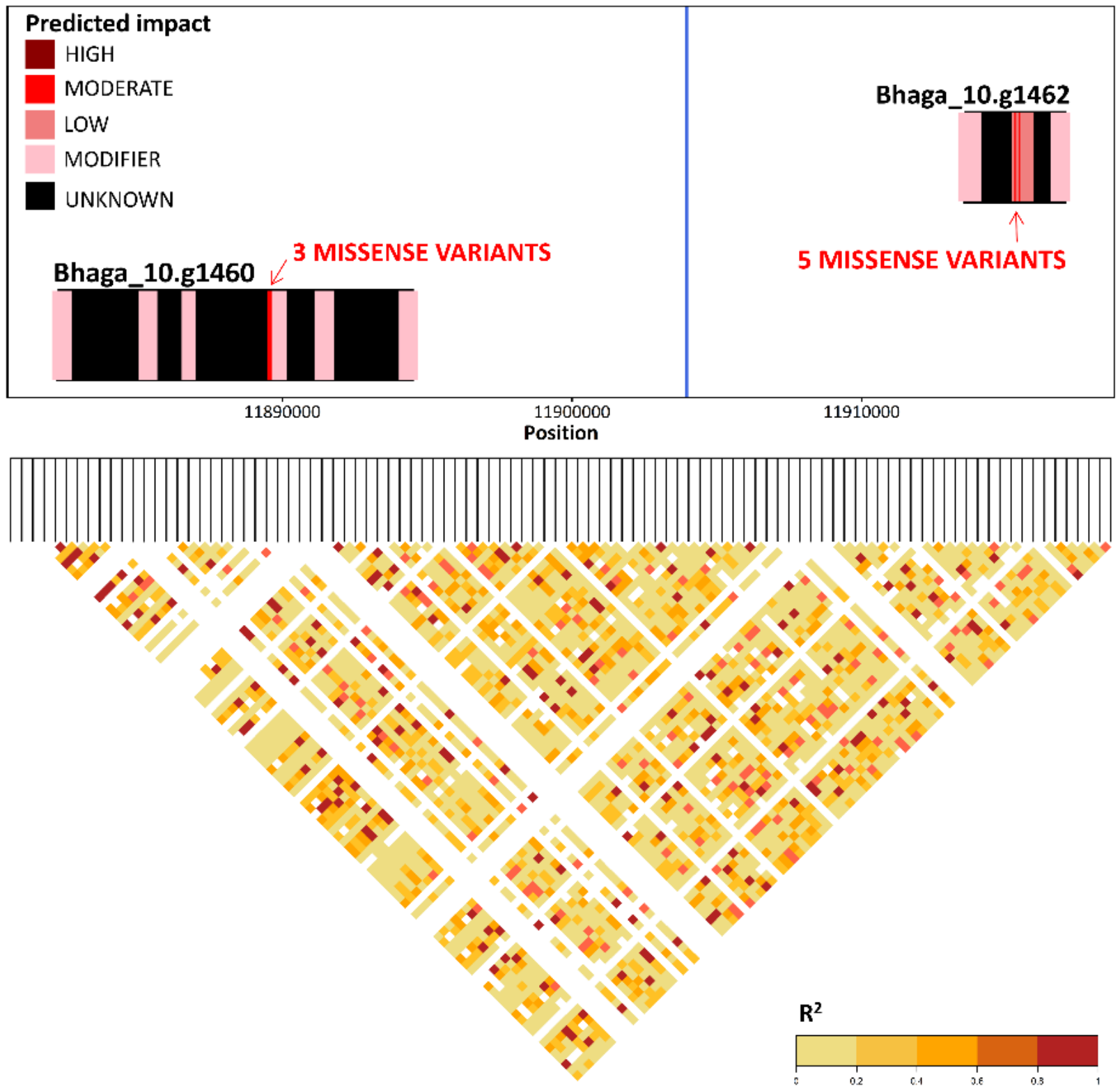

S16: Coding region of the gene variant *Bhaga_10.g1651 (BONZAI1), Bhaga_10.g1652* (uncharacterized) and the significant marker on chromosome 10 at ~ 13.44 Mb in blue and pairwise LD heat map from 13420289 to 13440289 Mb.

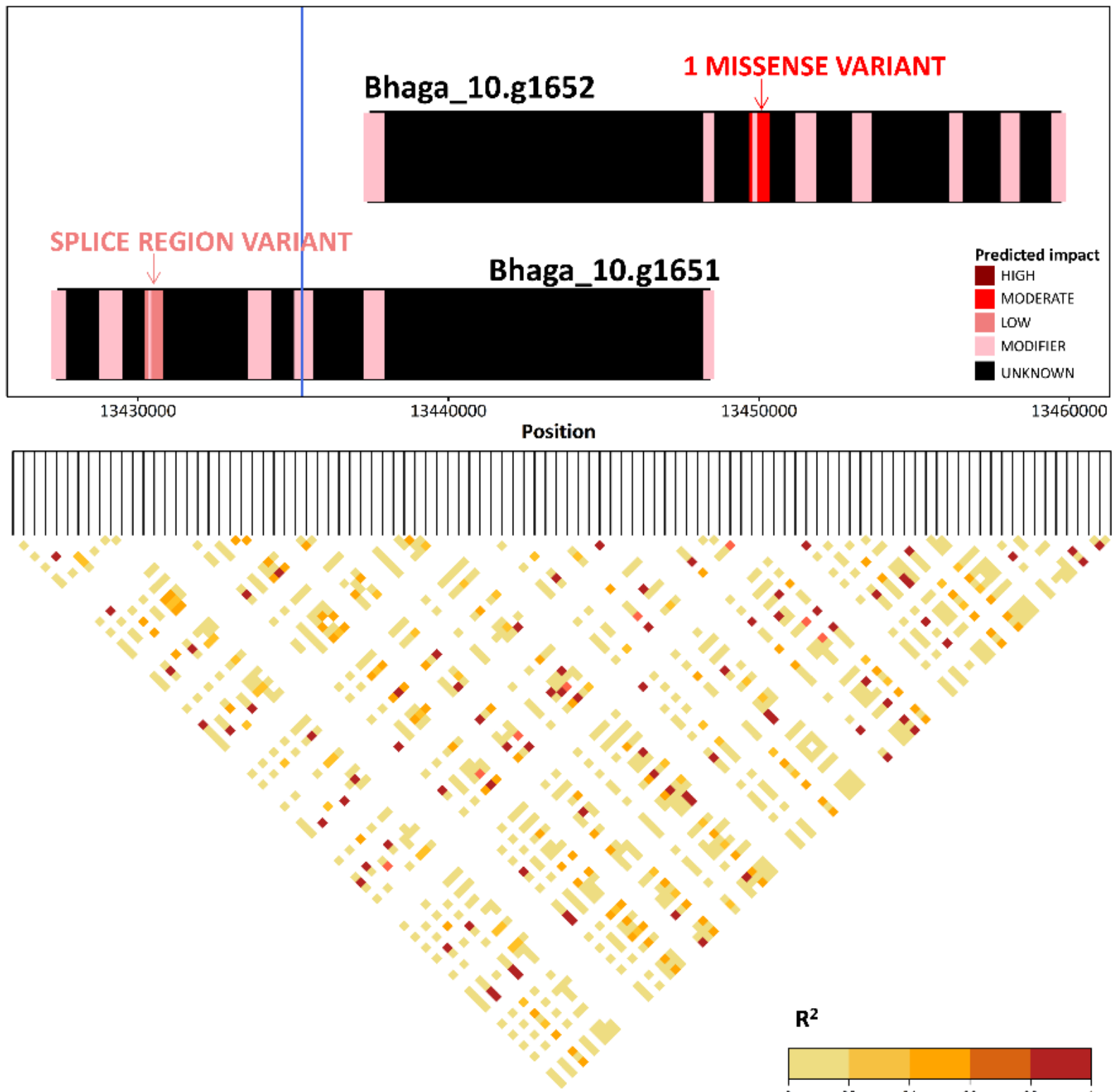

S17: List of other genes close to markers associated with stomatal density but without a direct impact on stomatal density based on the literature review

Gene variant name	Gene	Gene description based on the literature review
Bhaga_10.g867	**Capsanthin synthase chromoplastic-like**	Capsanthin synthase chromoplastic-like is mainly studied and observed in species of the *Capsicumin* genus like paprika, pepper or chili pepper (Piano et al. 2019) and carrot (Deng et al. 2024), but also found in some species of *Lilium leichtinii* (lily) (Valadon and Mummery 1977) and asparagus (Deli et al. 2000).
Bhaga_10.g873	**mRNA-decapping enzyme-like protein**	The gene mRNA-decapping enzyme-like protein regulates mRNA decapping (Vidya and Duchaine 2022). Decapping of the mRNA means the removal of the m7G cap, which leads quickly to mRNA degradation (Vidya and Duchaine 2022).
Bhaga_10.g1674	**poly(A) polymerase I-like gene**	The poly(A) polymerase I-like gene is responsible 3'polyadenylation which leads to rapid degradation of 3' to 5' exonucleases (Feng and Cohen 2000). This gene was so far only studied in E. coli (Feng and Cohen 2000).

References

Deli J, Z Matus, G Tóth et al. (2000) J Agric Food Chem 48:2793−2796. https://doi.org/10.1021/jf991243h

Deng YJ, Duan AQ, Liu H et al. (2024) Generation of high purity capsanthin and capsorubin through synthet-ic metabolic engineering in carrot germplasm. J Exp Bot 75:7202–7216. https://doi.org/10.1093/jxb/erae351

Feng Y, Cohen SN (2000) Unpaired terminal nucleotides and 5′ monophosphorylation govern 3′ polyadenyla-tion by Escherichia coli poly(A) polymerase I. Proc Natl Acad Sci USA: 6415–6420

Piano D, Cocco E, Guadalupi G et al. (2019) Characterization under quasi-native conditions of the cap-santhin/capsorubin synthase from Capsicum annuum L. Plant Physiol Biochem 143:165–175. https://doi.org/10.1016/j.plaphy.2019.09.007

Valadon L, Mummery RS (1977) Carotenoids of lilies and of red pepper: Biogenesis of Capsanthin and Capso-rubin. Zeitschrift für Pflanzenphysiologie 82:407–416. https://doi.org/10.1016/S0044-328X(77)80004-4

Vidya E, Duchaine TF (2022) Eukaryotic mRNA decapping activation. Front Genet 13:832547. https://doi.org/10.3389/fgene.2022.832547

S18: Minor allele frequency (MAF) observed in the different stands at the significant markers (p ≤0.000001) on chromosome 10 associated with stomatal density and the number of heterozygous individuals (Nr of Het), number of homozygous individuals (Nr of Hom) and the total number of genotyped individuals (Nr of total ind.).

**Marker**	**Stand**	**MAF**	**Nr of Het.**	**Nr of total ind.**	**Marker**	**MAF**	**Nr of Het**	**Nr of total ind.**
chr10_7198804	Lempes	0.015	3		chr10_10909306	0	0	100
chr10_7198804	Tampa	0.0354	7		chr10_10909306	0	0	99
chr10_7198804	Solomon	0.0357	7		chr10_10909306	0.0204	4	98
chr10_7198804	Lupului	0.1364	27		chr10_10909306	0.1224	22	98
chr10_7198804	Ruia	0.3838	52		chr10_10909306	0.4798	65	99
chr10_7211779	Lempes	0.0052	1		chr10_10909343	0	0	100
chr10_7211779	Tampa	0.0152	3		chr10_10909343	0	0	99
chr10_7211779	Solomon	0.0204	4		chr10_10909343	0.0204	4	98
chr10_7211779	Lupului	0.1042	20		chr10_10909343	0.1224	22	98
chr10_7211779	Ruia	0.3687	51		chr10_10909343	0.4798	65	99
chr10_7555433	Lempes	0	0	100	chr10_11233527	0.0054	1	93
chr10_7555433	Tampa	0.0101	2	99	chr10_11233527	0	0	97
chr10_7555433	Solomon	0.0102	2	98	chr10_11233527	0.0215	2	93
chr10_7555433	Lupului	0.102	20	98	chr10_11233527	0.1276	21	98
chr10_7555433	Ruia	0.4242	56	99	chr10_11233527	0.5808	45	99
chr10_7785927	Lempes	0	0	96	chr10_11233581	0.0053	1	94
chr10_7785927	Tampa	0.0102	2	98	chr10_11233581	0	0	97
chr10_7785927	Solomon	0.0103	2	97	chr10_11233581	0.0215	2	93
chr10_7785927	Lupului	0.1064	20	94	chr10_11233581	0.1276	21	98
chr10_7785927	Ruia	0.4242	56	99	chr10_11233581	0.5808	45	99
chr10_7787468	Lempes	0	0	84	chr10_11511420	0.0643	1	70
chr10_7787468	Tampa	0.0128	1	39	chr10_11511420	0.037	0	54
chr10_7787468	Solomon	0.0068	1	74	chr10_11511420	0.058	2	69
chr10_7787468	Lupului	0.0714	6	42	chr10_11511420	0.2041	4	49
chr10_7787468	Ruia	0.4026	42	77	chr10_11511420	0.7011	12	87
chr10_7820271	Lempes	0.0101	2	99	chr10_11903957	0.0165	3	91
chr10_7820271	Tampa	0.0532	10	94	chr10_11903957	0.0054	1	93
chr10_7820271	Solomon	0.0412	8	97	chr10_11903957	0.0054	1	92
chr10_7820271	Lupului	0.1474	26	95	chr10_11903957	0.1292	21	89
chr10_7820271	Ruia	0.4286	56	98	chr10_11903957	0.5312	56	96
chr10_7820389	Lempes	0	0	100	chr10_13435289	0	0	72
chr10_7820389	Tampa	0.0101	2	99	chr10_13435289	0.0204	2	49
chr10_7820389	Solomon	0.0102	2	98	chr10_13435289	0.0211	3	71
chr10_7820389	Lupului	0.102	20	98	chr10_13435289	0.1071	7	42
chr10_7820389	Ruia	0.4242	56	99	chr10_13435289	0.2722	31	79
chr10_10909126	Lempes	0	0	95	chr10_13677756	0	0	64
chr10_10909126	Tampa	0	0	85	chr10_13677756	0	0	55
chr10_10909126	Solomon	0.0217	4	92	chr10_13677756	0.0086	1	58
chr10_10909126	Lupului	0.1312	19	80	chr10_13677756	0.0581	5	43
chr10_10909126	Ruia	0.4899	65	99	chr10_13677756	0.3725	8	51
chr10_10909168	Lempes	0	0	95				
chr10_10909168	Tampa	0	0	91				
chr10_10909168	Solomon	0.0204	4	98				
chr10_10909168	Lupului	0.1359	21	92				
chr10_10909168	Ruia	0.4848	64	99				

S19: Correlation between stomatal density and the minor allele frequency (MAF) of the significant markers on chromosome 10 at ~7.116 (a), ~ 7.199 (b), ~7.212 (c), ~7.555 (d), ~7.7859 (e), ~7.82 (f), (g), ~11.9 (h), ~13.67776 (i).

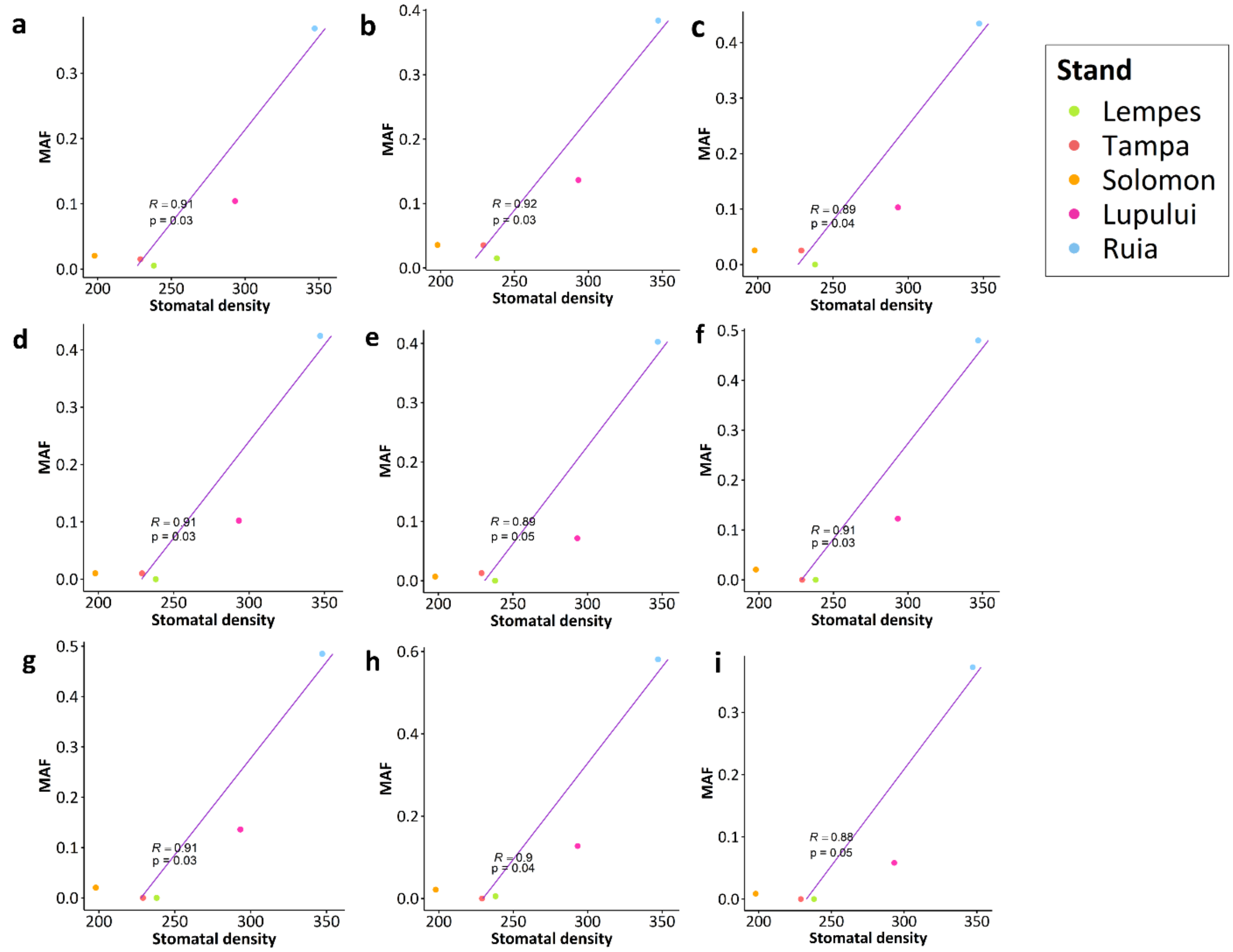

S20: Minor allele frequency (MAF) observed in the different stands at the significant markers (p ≤0.000001) associated with leaf nitrogen content, the stand mean, the number of heterozygous individuals (Nr of Het) and the total number of genotyped individuals (Nr of total ind.).

Marker	Population	MAF	Nr of Het.	Nr of total ind	Leaf nitrogen content (2020)	Leaf nitrogen content (2021)	Leaf nitrogen content (2022)
chr1_38020777	Lempes	0.014851	3	101	2.086535	2.910145	2.830541
chr1_38020777	Tampa	0.055556	11	99	2.077564	2.885671	2.744564
chr1_38020777	Solomon	0.059406	12	101	2.009534	2.577074	2.519506
chr1_38020777	Lupului	0.064356	13	101	2.211398	2.708902	2.732698
chr1_38020777	Ruia	0.045872	10	109	2.203298	2.933279	2.856909

S21: Correlation between leaf nitrogen content measured in 2021 and the minor allele frequency (MAF) of the significant marker (p ≤0.000001) on chromosome 2 at ~31.804 Mb. This marker was annotated with the gene variant *Bhaga_2.g3457* with the underlying gene *XP_030960280.1,* also known as *abscisic-aldehyde oxidase-like*.

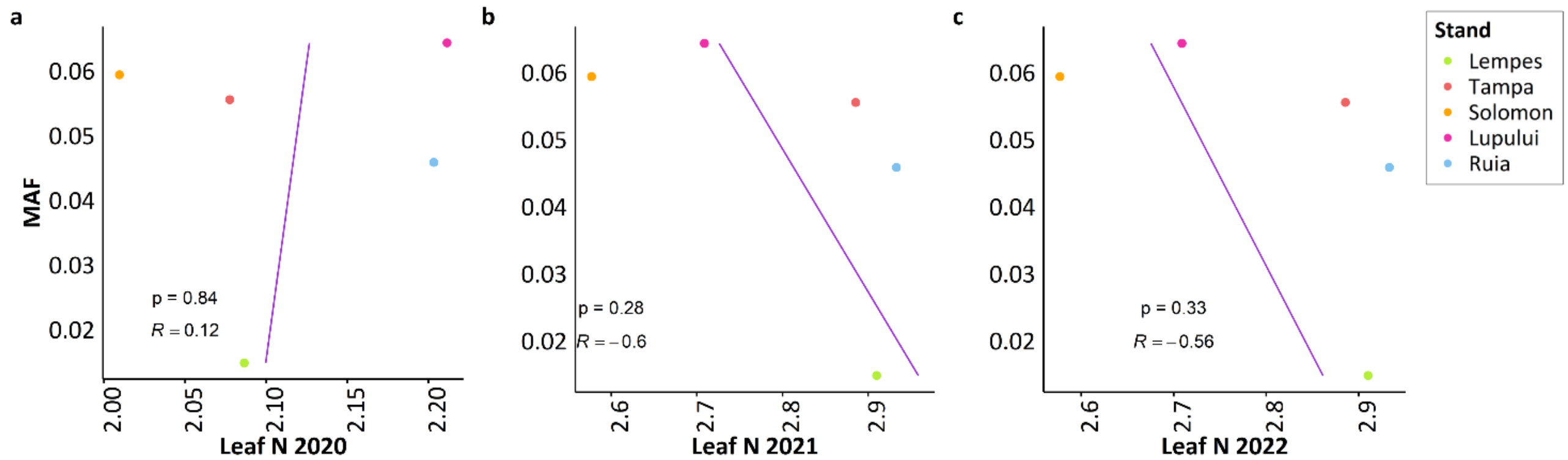

S22: Rate of false positive observations observed in Ghat test calculated based on the 100 randomly generated traits with 10 replications (10 replications x 100 random traits).

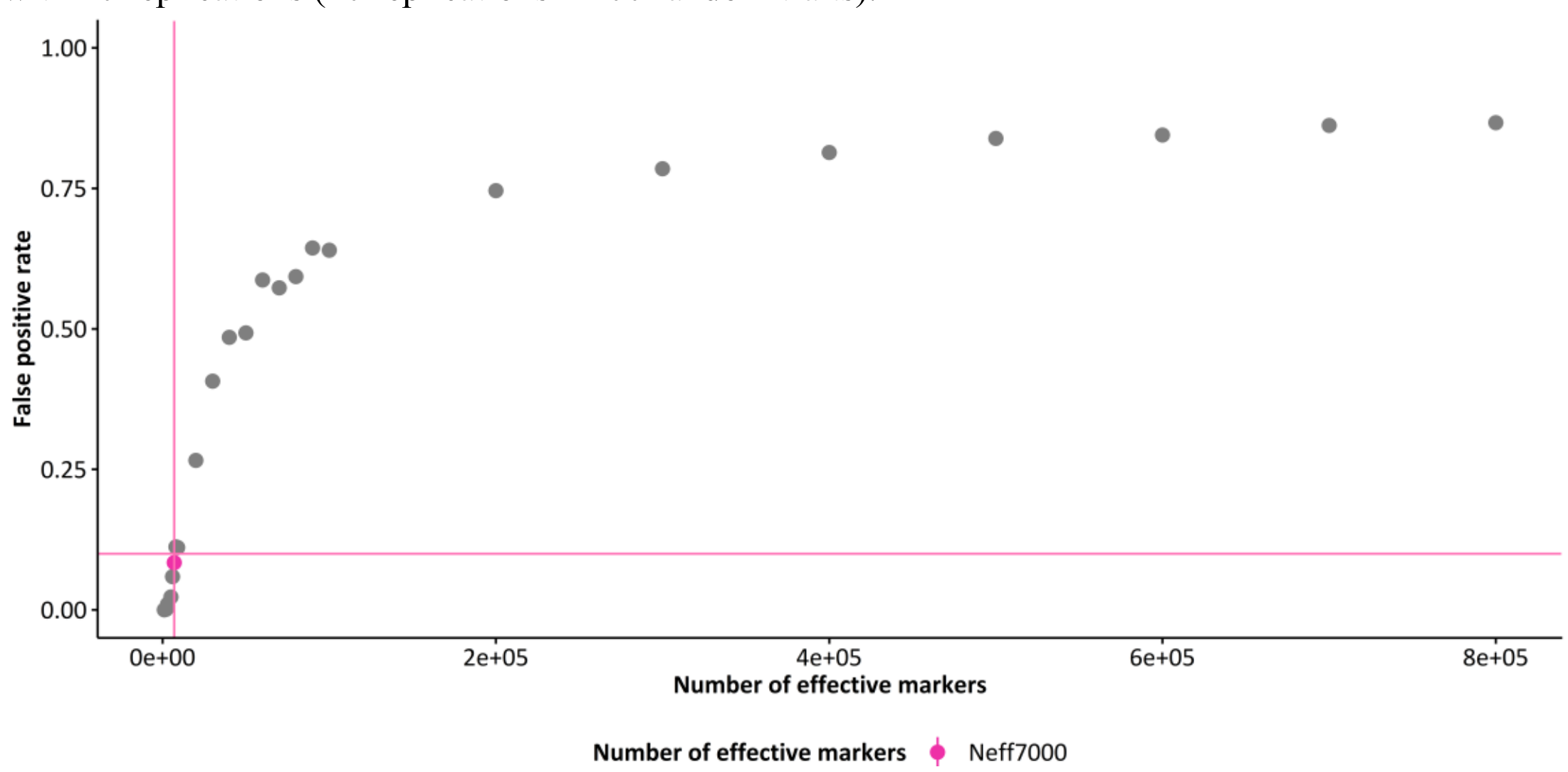

S23: Results from five-fold cross-validation with the mean predictive ability as $r_{y\hat{g}}$ (correlation of predicted trait estimates and observed trait measurements) with random partition of stands in test and training set (**a**) and five-fold cross-validation in which one stand was left out and the remaining stands were used for prediction (**b**) with 100 replications based on RR-BLUP for the real trait measurements.

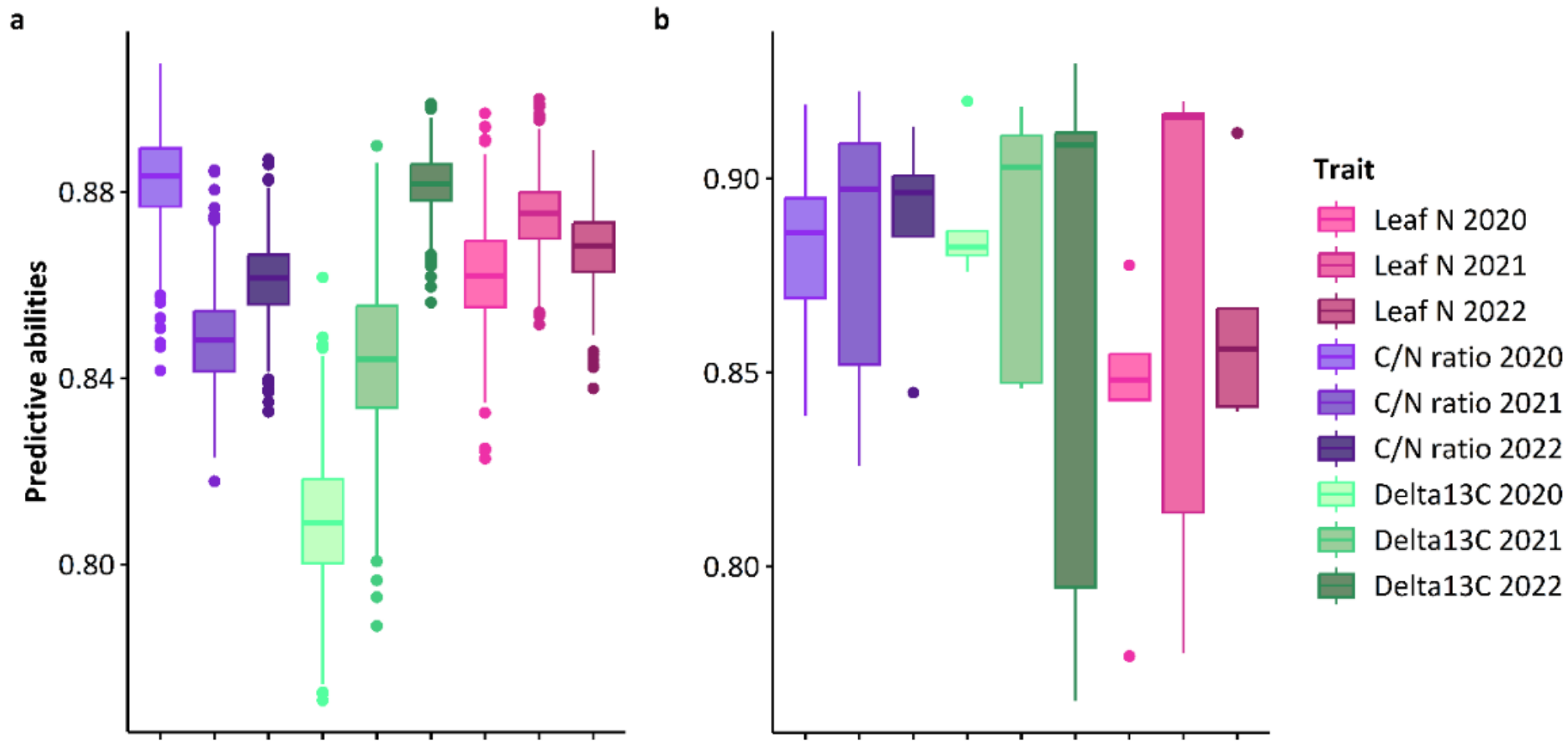

S24: Results from $\widehat{G}$ test with change calculated between groups (Ruia, Lupului vs Tampa, Lempes) calculated between groups with 100000 permutations and 7000 effective markers calculated based on the previous test runs for carbon isotope composition $\delta^{13}C$.

**Trait**	**$\widehat{G}$ correlation coefficient**	**p-value**
$\delta^{13}C$ (2020)	0.1017	0.0000
$\delta^{13}C$ (2021)	0.0962	0.0000
$\delta^{13}C$ (2022)	-0.017	0.1527
Leaf N content (2020)	-0.0105	0.3698
Leaf N content (2021)	-0.0383	0.0014
Leaf N content (2022)	-0.0188	0.1279
C/N ratio (2020)	0.0215	0.0623
C/N ratio (2021)	0.0289	0.0192
C/N ratio (2022)	0.0105	0.3866

S25: Observed codon changes which lead to an amino acid (AA) replacement and a resulting change in polar bonds (hydrophobic or hydrophilic) at the underlying gene variants of markers associated with stomatal density

**Gene variant**	**Variant version**	**Change in polar bonds**	**Original**	**Replaced by**
Bhaga_8.g506	5 missense variants	Hydrophobic AA was replaced by hydrophilic AA	Pro	Ser
		Hydrophilic AA was replaced by hydrophobic AA	Glu	Asp
		Hydrophilic AA was replaced by hydrophobic AA	Arg	Lys
		Hydrophilic AA was replaced by hydrophobic AA	Val	Ala
		Hydrophobic AA was replaced by hydrophilic AA	Asn	Lys
		Hydrophilic AA was replaced by hydrophobic AA	Glu	Asp
	3 missense variants	no change in polar bonds		
Bhaga_8.g1863	1 missense variant	Hydrophilic AA was replaced by hydrophobic AA	Lys	Asn
Bhaga_9.g1680	1 missense variant	Hydrophilic AA was replaced by hydrophobic AA	Asp	His
Bhaga_9.g1682	13 missense variants	Hydrophobic AA was replaced by hydrophilic AA	Asn	Lys
		Hydrophilic AA was replaced by hydrophobic AA	Arg	Tyr
		Hydrophilic AA was replaced by hydrophobic AA	Asn	Ile
		Hydrophilic AA was replaced by hydrophobic AA	Lys	Glu
		Hydrophilic AA was replaced by hydrophobic AA	Glu	Asp
		Hydrophilic AA was replaced by hydrophobic AA	Thr	Ser
		Hydrophobic AA was replaced by hydrophilic AA	Tyr	Asp
		Hydrophilic AA was replaced by hydrophobic AA	Lys	Glu

		Hydrophilic AA was replaced by hydrophobic AA	Arg	Gly
		Hydrophobic AA was replaced by hydrophilic AA	Gly	Asp
		Hydrophobic AA was replaced by hydrophilic AA	Asn	Ser
		Hydrophilic AA was replaced by hydrophobic AA	His	Leu
		Hydrophilic AA was replaced by hydrophobic AA	Gly	Val
Bhaga_10.g591	3 missense variants	Hydrophobic AA was replaced by hydrophilic AA	Leu	His
		Hydrophobic AA was replaced by hydrophilic AA	Asn	His
		Hydrophilic AA was replaced by hydrophobic AA	Leu	Val
Bhaga_10.g752	1 missense variant	Hydrophilic AA was replaced by hydrophobic AA	Glu	Lys
Bhaga_10.g753	3 missense variants	Hydrophilic AA was replaced by hydrophobic AA	Val	Ile
		no change in polar bonds	Gln	Lys
		Hydrophobic AA was replaced by hydrophilic AA	Met	Lys
Bhaga_10.g852	1 missense variant	Hydrophilic AA was replaced by hydrophobic AA	Val	Ile
Bhaga_10.g854	7 missense variants	Hydrophilic AA was replaced by hydrophobic AA	Phe	Tyr
		Hydrophilic AA was replaced by hydrophobic AA	Leu	Phe
		Hydrophilic AA was replaced by hydrophobic AA	Ala	Val
		Hydrophobic AA was replaced by hydrophilic AA	Asn	Lys
		Hydrophilic AA was replaced by hydrophobic AA	Val	Ile
		Hydrophilic AA was replaced by hydrophobic AA	Glu	Gly
		Hydrophilic AA was replaced by hydrophobic AA	Phe	Val
Bhaga_10.g867	7 missense variants	Hydrophilic AA was replaced by hydrophobic AA	Thr	Asn
		Hydrophilic AA was replaced by hydrophobic AA	Leu	Phe
		Hydrophilic AA was replaced by hydrophobic AA	Gly	Ala
		Hydrophilic AA was replaced by hydrophobic AA	His	Leu
		Hydrophilic AA was replaced by hydrophobic AA	Ser	Trp
		Hydrophobic AA was replaced by hydrophilic AA	Asn	His
		Hydrophobic AA was replaced by hydrophilic AA	Asn	Lys
Bhaga_10.g917	9 missense variants	Hydrophilic AA was replaced by hydrophobic AA	Phe	Leu
		Hydrophilic AA was replaced by hydrophobic AA	Asp	Ala
		Hydrophilic AA was replaced by hydrophobic AA	Ser	Cys
		Hydrophobic AA was replaced by hydrophilic AA	Leu	Phe
		Hydrophobic AA was replaced by hydrophilic AA	Ile	Ser
		Hydrophobic AA was replaced by hydrophilic AA	Gly	Glu
		Hydrophobic AA was replaced by hydrophilic AA	Gly	Arg
		Hydrophilic AA was replaced by hydrophobic AA	Ser	Leu
		Hydrophilic AA was replaced by hydrophobic AA	Ser	Asn
Bhaga_10.g957	5 missense variants	Hydrophilic AA was replaced by hydrophobic AA	Glu	Lys
		Hydrophobic AA was replaced by hydrophilic AA	Asn	Lys
		Hydrophobic AA was replaced by hydrophilic AA	Pro	Thr
		no change in polar bonds	Ala	Val
		Hydrophilic AA was replaced by hydrophobic AA	Arg	Gln
	25 missense variants	no change in polar bonds		
Bhaga_10.g1460	2 missense variants	Hydrophilic AA was replaced by hydrophobic AA	Gly	Ala
		Hydrophilic AA was replaced by hydrophobic AA	Ile	Val
	1 missense variant	no change in polar bonds		
Bhaga_10.g1462	5 missense variants	Hydrophobic AA was replaced by hydrophilic AA	Ala	Ser
		Hydrophilic AA was replaced by hydrophobic AA	Thr	Lys
		Hydrophobic AA was replaced by hydrophilic AA	Gly	Arg
		Hydrophilic AA was replaced by hydrophobic AA	Asp	Asn
		Hydrophilic AA was replaced by hydrophobic AA	Asp	Glu
Bhaga_10.g1543	3 missense variants	Hydrophobic AA was replaced by hydrophilic AA	Tyr	His
		Hydrophobic AA was replaced by hydrophilic AA	Phe	Cys
		Hydrophilic AA was replaced by hydrophobic AA	Ser	Thr
Bhaga_10.g1652	1 missense variant	Hydrophilic AA was replaced by hydrophobic AA	Glu	Lys
Bhaga_10.g1674	5 missense variants	Hydrophilic AA was replaced by hydrophobic AA	Ile	Leu
		Hydrophilic AA was replaced by hydrophobic AA	Val	Ala
		Hydrophilic AA was replaced by hydrophobic AA	Arg	Pro
		Hydrophilic AA was replaced by hydrophobic AA	Arg	Gly
		Hydrophobic AA was replaced by hydrophilic AA	Leu	His

S26: Genes and their functional categories of genes derived from the KEGG database (2025) and the level of significance shown as -$\log_{10}$(p) of the underlying SNP markers from the GWAS for stomatal density.

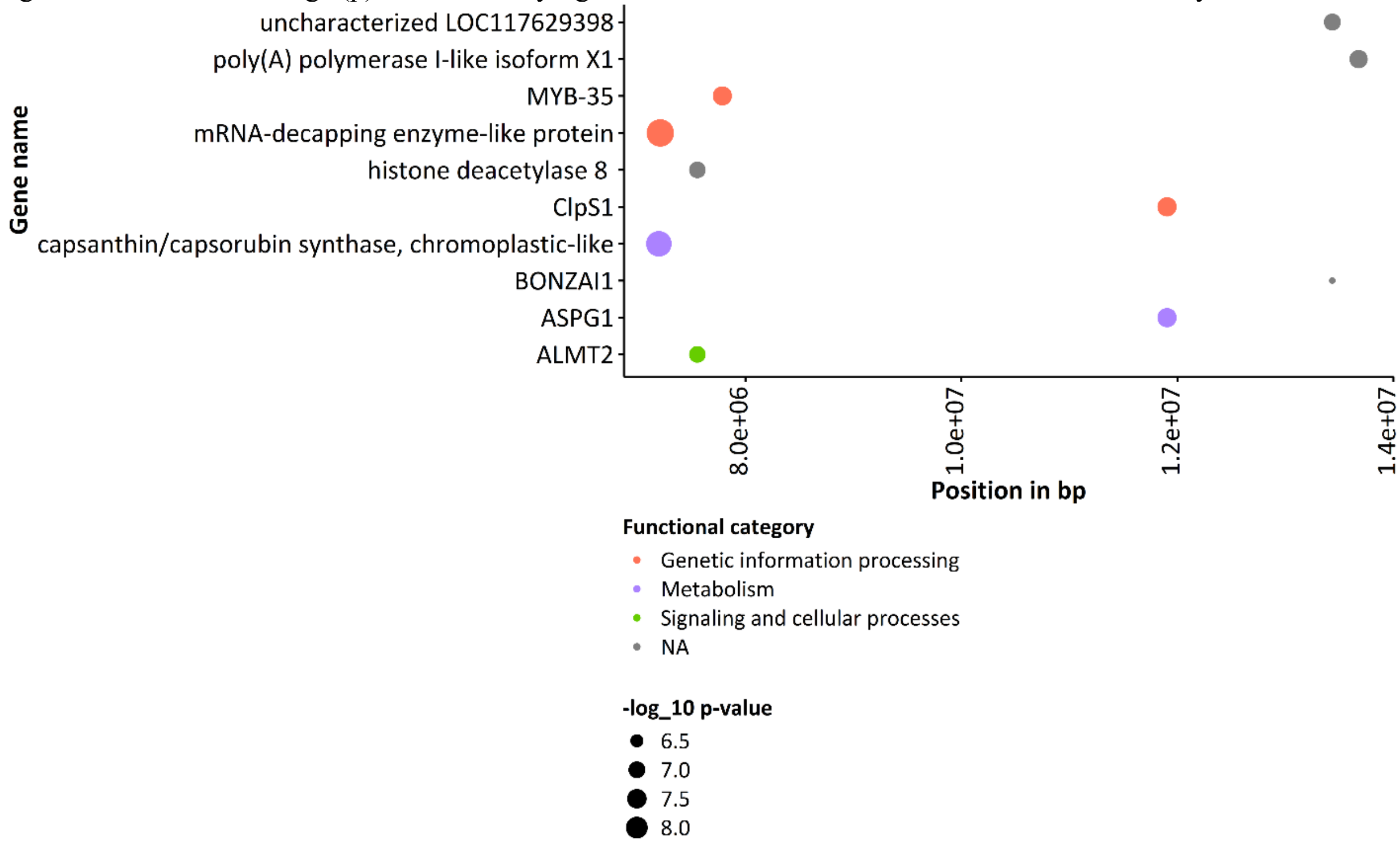

S27: Correlation between stomatal density and observed genotypes at the significant marker (p ≤0.000001) on chromosome 10 at ~7.82 Mb. The coefficient of determination R^2 was 0.2353 (p ~0). This marker is close to the coding region of the gene variant *Bhaga_10.g957* (*MYB35-like*)

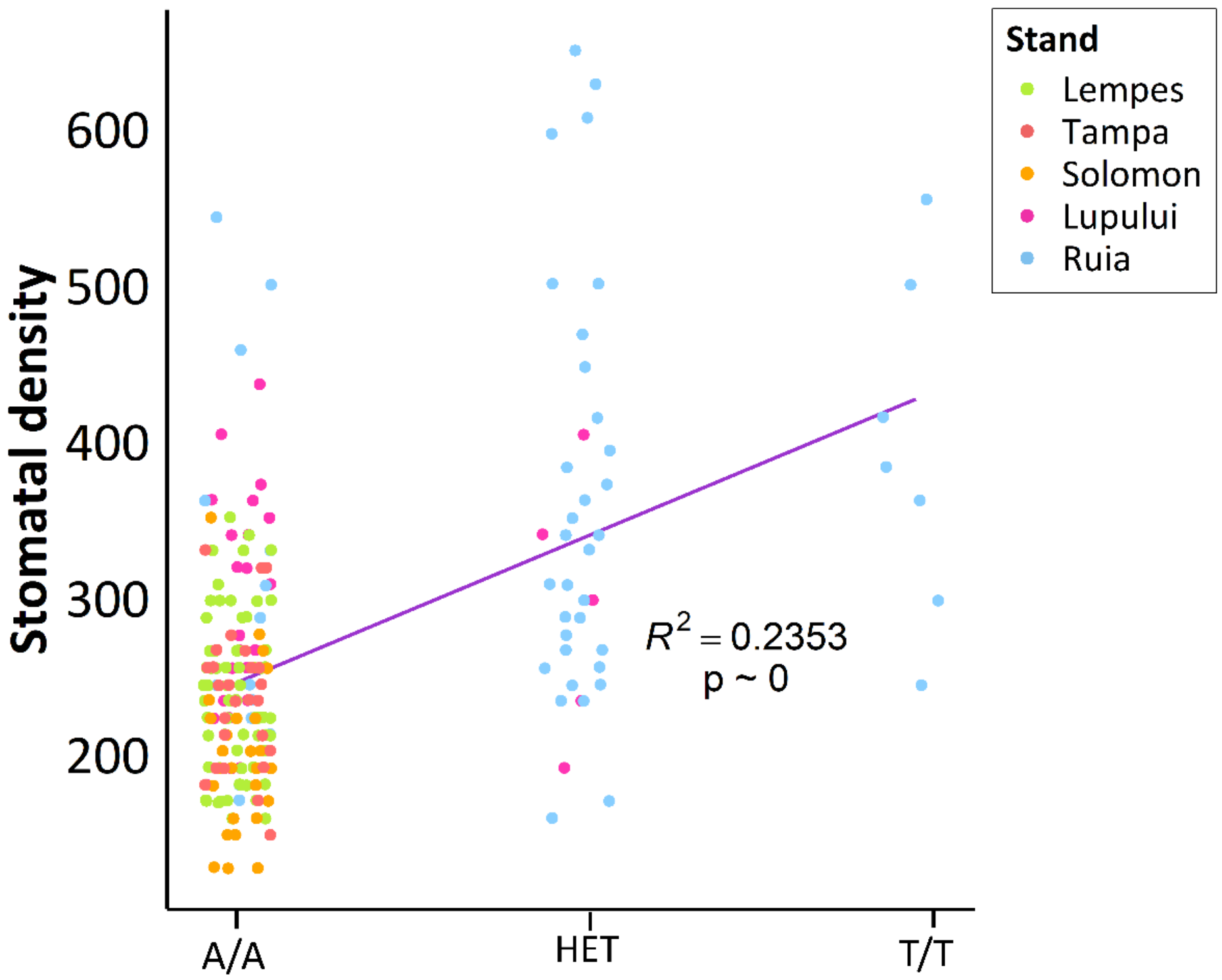

Supplement: Supplementary file 1 [file PPL-178-e71120-s001.pdf]
